# Cardiomyocyte-intrinsic somatic mtDNA mutations induce an OXPHOS-dependent immune response and promote progressive heart failure

**DOI:** 10.1126/sciadv.aec8606

**Published:** 2026-09-04

**Authors:** Kristina Bubb, Giovanni Rigoni, Polyxeni Papadea, Gianluigi Pironti, Jelena Misic, Shan Jiang, David Alsina, Diana Rubalcava-Gracia, Daniel C. Andersson, Roberta Filograna, Camilla Koolmeister, Patrick Giavalisco, Anna Wredenberg, Matthias Mann, Florian A. Rosenberger, Nils-Göran Larsson

**Affiliations:** ^1^ Department of Medical Biochemistry and Biophysics, Karolinska Institutet, Stockholm, Sweden.; ^2^ Department of Physiology and Pharmacology, Karolinska Institutet, Stockholm, Sweden.; ^3^ Department of Medicine Solna, Cardiology Research Unit, Karolinska Institutet, Stockholm, Sweden.; ^4^ School of Life Sciences, Technical University of Munich, Freising, Germany.; ^5^ Center for Inherited Metabolic Diseases, Karolinska University Hospital, Stockholm, Sweden.; ^6^ Departamento de Biología Molecular y Biotecnología, Instituto de Investigaciones Biomédicas, Universidad Nacional Autónoma de México, Mexico City, México.; ^7^ Cardiology Unit Heart and Vascular Theme, Karolinska University Hospital, Stockholm, Sweden.; ^8^ Metabolomics Core Facility, Max Planck Institute for Biology of Ageing, Cologne, Germany.; ^9^ Proteomics and Signal Transduction, Max Planck Institute of Biochemistry, Martinsried, Germany.; ^10^ Department of Medical Biochemistry and Biophysics, Science for Life Laboratory, Karolinska Institutet, Stockholm, Sweden.

## Abstract

Mitochondrial DNA (mtDNA) mutations accumulate with age, but their mechanistic contribution to aging remains unclear. The classical mtDNA mutator mouse expresses a proofreading-deficient mtDNA polymerase (POLG^D257A^) and accumulates mtDNA mutations across all tissues leading to premature aging. However, this model cannot resolve whether the aging phenotype results from systemic dysfunction or cell-intrinsic effects of somatic mtDNA mutations. To overcome this limitation, we generated *Polg*^iMut^ mice allowing spatial and temporal control of POLG^D257A^ expression. We demonstrate here that mtDNA mutations induced in cardiomyocytes cause progressive contractile dysfunction and respiratory chain deficiency in the heart without accompanying systemic pathology. Proteomic analyses link cardiac mosaic respiratory chain dysfunction to a progressive immune response, characterized by up-regulation of antigen-processing proteins and immune cell infiltration. In contrast, longevity-associated pathways are suppressed and uncoupled from mitochondrial and immune alterations, indicating distinct regulatory mechanisms. These findings demonstrate that mtDNA mutations can drive cardiac dysfunction and reveal a mechanistic link between mitochondrial dysfunction, immune responses, and aging.

## INTRODUCTION

Mitochondrial dysfunction is a recognized hallmark of aging, fundamentally contributing to the progressive decline of tissue homeostasis and the increased incidence of age-related diseases (*1*–*4*). Among the diverse mitochondrial alterations documented in aging, the accumulation of somatic mutations in mitochondrial DNA (mtDNA) is one of the most consistently documented features across mammalian species, including humans (*5*, *6*). Most mtDNA mutations are generated by replication errors, whereas oxidative damage is less important (*7*, *8*). The mtDNA is extensively replicated during embryonic development and is continuously turned over also in postmitotic tissues (*9*, *10*), which explains why mtDNA exhibits a mutation rate up to 100-fold higher than that of nuclear DNA (*5*, *8*). Mutations of mtDNA can be maternally inherited or arise de novo during development or postnatal life (*11*) and can undergo clonal expansion leading to mosaic oxidative phosphorylation (OXPHOS) dysfunction in aging mammalian tissues (*6*, *12*–*14*).

The development of the mtDNA mutator mouse (henceforth denoted *Polg*^Mut^ mice) with homozygous expression of a proofreading-deficient mtDNA polymerase γ (POLG^D257A^) provided direct genetic evidence that accumulation of mtDNA mutations alone is sufficient to induce premature aging (*15*, *16*). The *Polg*^Mut^ mice accumulate random mtDNA mutations and develop early-onset aging phenotypes, including reduced lifespan, cardiomyopathy, kyphosis, hearing loss, and anemia, coupled with OXPHOS dysfunction in various tissues (*15*, *17*, *18*). Over the past two decades, the *Polg*^Mut^ mouse model has been instrumental in demonstrating how mtDNA mutations can accelerate aging, revealing proposed downstream mechanisms including stem cell exhaustion (*19*), metabolic reprogramming (*20*), nucleotide imbalance (*21*, *22*), and chronic inflammation (*23*–*25*) as possible drivers of premature aging.

Inflammation has emerged as a critical mediator linking mtDNA mutations to aging. In the mtDNA mutator mouse, mtDNA has been reported to be released into the cytosol where it functions as a damage-associated molecular pattern. This release, in turn, activates the cGAS-STING pathway to elicit a robust type I interferon (IFN-I) response and chronic inflammation that mirror hallmarks of “inflammaging” (*23*). This proinflammatory milieu drives systemic elevation of cytokines such as TNFα, MCP-1, Il5, and CCL2 and expansion of inflammatory myeloid populations (*23*, *26*), collectively contributing to accelerated organismal aging of these mice.

While innate immune activation via cytosolic mtDNA sensing is well established, recent evidence suggests that the mitochondrial genome may also engage the adaptive immune system. Sustained IFN-I signaling can up-regulate components of the antigen presentation machinery, thereby enhancing the ability to present peptides via major histocompatibility complex (MHC) molecules on the cell surface (*27*–*29*). In parallel, mutated mitochondrial peptides can be exported to the cytosol and presented as neoantigens on the cell surface to trigger T cell responses (*30*, *31*). These processes, although mechanistically independent of cGAS-STING signaling, may converge functionally to amplify the immune activation. Thus, mtDNA mutations act not only as drivers of mosaic OXPHOS dysfunction but are also potent triggers of immunogenic stress, altering immune surveillance and bridging innate and adaptive immune responses across tissues and thereby potentially accelerating tissue dysfunction and age-associated decline.

Despite the valuable mechanistic insights yielded by the *Polg*^Mut^ mouse model, key limitations remain. The widespread accumulation of mtDNA mutations across diverse cell types complicates the distinction between cell-intrinsic effects and secondary responses driven by systemic dysfunction. Moreover, the mtDNA mutator phenotype is present already in the early embryo (*32*) and may cause developmental aberrations (*11*) that contribute to the phenotypes in adult mice. Previous efforts to develop inducible mtDNA mutator models have failed to generate sufficient mutation burdens or to recapitulate the *Polg*^Mut^ mouse phenotypes (*33*, *34*). As a result, we still lack a clear understanding of how mtDNA mutations initiate organ-specific dysfunction and drive age-related pathologies.

To overcome these limitations, we developed an inducible mtDNA mutator mouse model (*Polg*^iMut^ mice) that enables spatial and temporal control of mtDNA mutagenesis through cre-loxP–mediated activation of POLG^D257A^ expression. Mice with homozygous whole-body activation of the inducible allele (*Polg*^wMut^ mice) develop a phenotype that is very similar to the classical *Polg*^Mut^ phenotype, thus validating that the inducible allele is functional. We proceeded to use this model to induce mtDNA mutagenesis in the heart (*Polg*^hMut^ mice) and show here that mtDNA mutation accumulation in cardiomyocytes leads to early contractile dysfunction, likely driven by mosaic OXPHOS dysfunction, preceding overt proteomic remodeling. Quantitative proteomic analysis revealed an initial metabolic adaptation marked by increased abundance of cytochrome P450 and amino acid metabolism enzymes in response to the rising mutation burden. As the number of mtDNA mutations increased further, a robust immunogenic signature emerged, including up-regulation of MHC class II antigen presentation pathways, IFN responses, and immune cell recruitment into the heart. This immunogenic signature closely correlated with mtDNA mutation burden and OXPHOS subunit depletion, whereas pathways associated with longevity regulation showed no such correlation and were uniformly down-regulated across all mutant hearts. These findings establish a direct link between cell-intrinsic mtDNA mutations, mitochondrial OXPHOS dysfunction, and immune responses, while showing that repression of longevity-associated pathways may not be directly triggered by mitochondrial dysfunction.

## RESULTS

### An inducible mtDNA mutator mouse model allowing spatial and temporal control of somatic mutagenesis of mtDNA

**Fig. 1.**
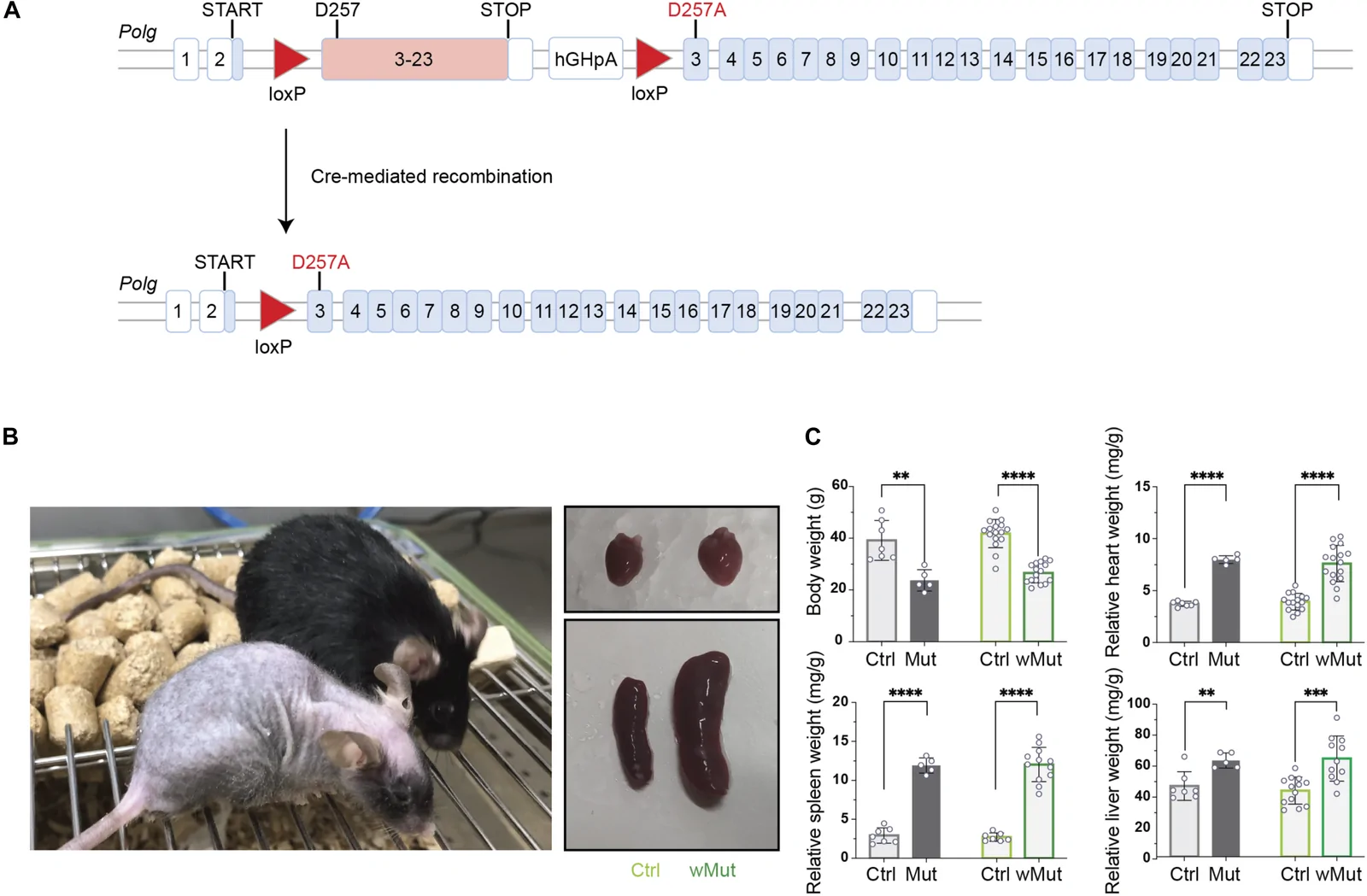
An inducible mtDNA mutator knock-in mouse model (*Polg*^iMut^). (**A**) Schematic representation of the inducible *Polg*^iMut^ knock-in allele. The locus contains a floxed wild-type *Polg* cDNA (exons 3 to 23) followed by a human growth hormone polyadenylation signal (hGHpA). This cassette prevents expression of the downstream mutant exon 3 encoding the D257A substitution. Upon cre-mediated recombination, the wild-type cassette is excised, enabling expression of the POLG^D257A^ polymerase. (A) Created in BioRender. Larsson, N. (2026) https://BioRender.com/98chy93. (**B**) Phenotypic presentation and morphology of heart and spleen from control (Ctrl) and whole-body *Polg*^wMut^ (wMut) mice at the age of 9 months. The hairless mouse in the foreground is a wMut, while the mouse in the background is a Ctrl. (**C**) Quantification of body weight and organ weight (normalized to body weight; milligrams per gram) comparing the *Polg*^wMut^ mouse model (green) to the classical *Polg*^Mut^ mouse model (gray) at the age of 9 months. Data are presented as means ± SD, unpaired two-tailed Welch’s *t* test; **P < 0.01; ***P < 0.001; ****P < 0.0001.

To overcome the systemic and constitutive limitations of the *Polg*^Mut^ model (*15*), we generated an inducible knock-in mouse (*Polg*^iMut^) enabling cre-recombinase–dependent expression of a proofreading-deficient mtDNA polymerase (Fig. 1A). The *Polg*^iMut^ allele was engineered by inserting a floxed wild-type cDNA fragment (exons 3 to 23 of *Polg*) followed by a transcriptional stop cassette (human growth hormone polyadenylation signal, hGHpA) into intron 2 of the endogenous *Polg* locus. This knock-in cassette is positioned upstream of a mutated exon 3 (GAC > GCC) that allows the expression of POLG^D257A^, which is well documented to have a drastically decreased proofreading activity during mtDNA replication (*15*). Upon cre-mediated recombination, the inserted cassette is excised, enabling transcriptional read-through and activation of POLG^D257A^ expression (Fig. 1A).

To validate the functionality of the *Polg*^iMut^ allele, we performed crosses with β-actin–cre mice to obtain homozygous mice with whole-body expression of POLG^D257A^ (*Polg*^wMut^ mice; fig. S1A). By 9 months of age, the *Polg*^wMut^ mice exhibited hallmark features of the classical *Polg*^Mut^ mice, including kyphosis, alopecia, reduced body weight, marked enlargement of the heart and spleen, and increased liver–to–body weight ratio (Fig. 1, B and C). These results validate that the *Polg*^wMut^ mice functionally recapitulate key phenotypes seen in the classical mtDNA mutator mice.

### Somatic mtDNA mutation accumulation drives distinct tissue-specific proteomic responses

**Fig. 2.**
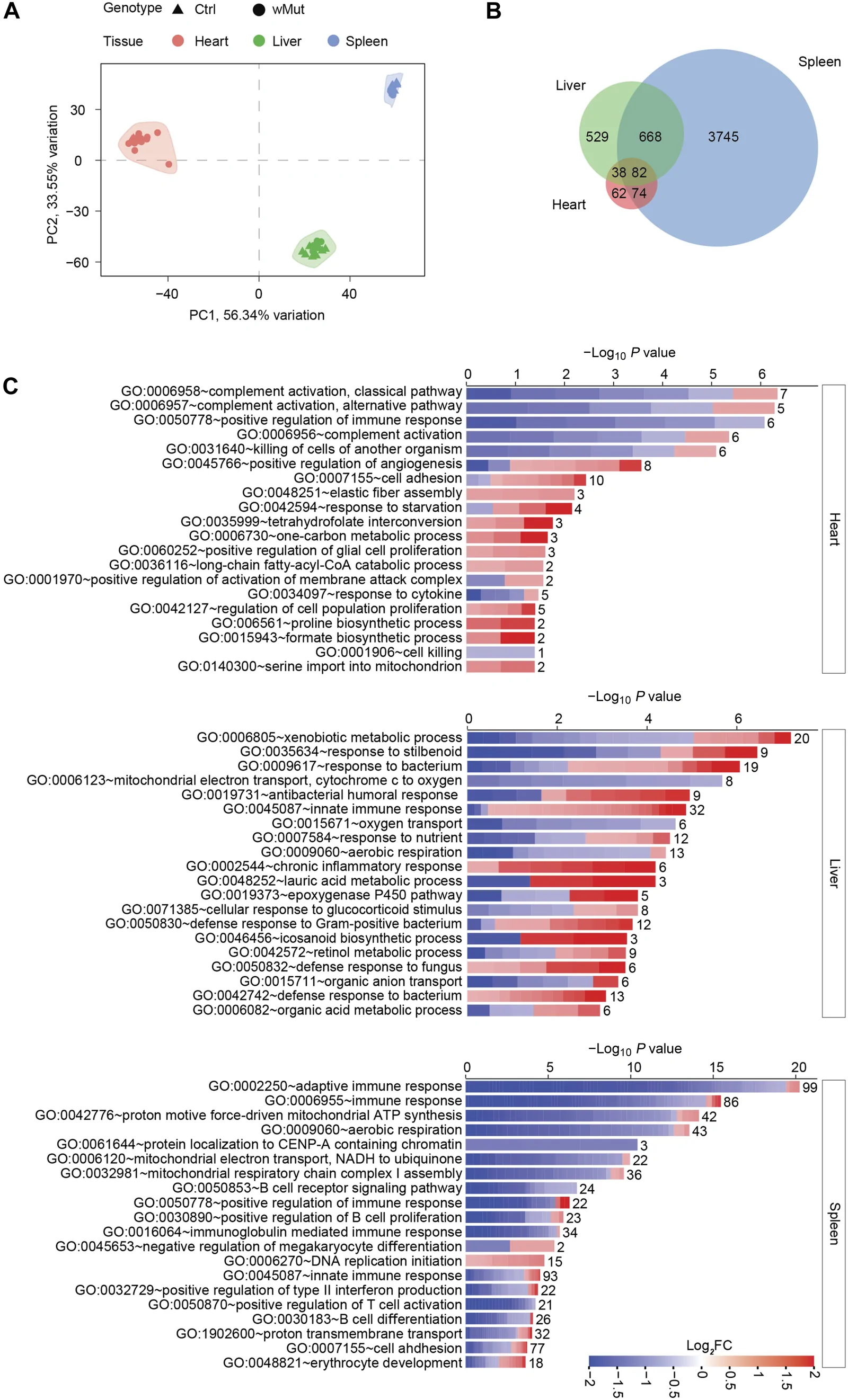
Tissue-specific proteomic responses to whole-body mtDNA mutation induction. (**A**) PCA of proteomic profiles from the heart, liver, and spleen of 9-month-old control and *Polg*^wMut^ mice. (**B**) Venn diagram showing the overlap among significantly altered protein abundances (−log_10_ adjusted *P* value >1.3) in the heart, liver, and spleen of 9-month-old *Polg*^wMut^ mice. (**C**) Functional enrichment analysis (DAVID) of differentially abundant proteins in heart, liver, and spleen (log_2_FC < −0.58 / > 0.58; −log_10_ adjusted *P* value >1.3) of *Polg*^wMut^ mice. Cell plots display the top 20 enriched pathways for each tissue, with up-regulated terms in red and down-regulated in blue. The number of annotated proteins per pathway is indicated next to the bar.

To understand how mtDNA mutations affect the proteome in a tissue-specific manner, we performed label-free quantitative proteomics on heart, liver, and spleen from 9-month-old *Polg*^wMut^ mice. Principal components analysis (PCA) confirmed that tissue identity was the primary source of protein expression variance, with samples clustering primarily by tissue type (Fig. 2A). Proteomic remodeling was most pronounced in the spleen, with 4569 proteins showing significant [false discovery rate (FDR) >0.05] changes in abundance, compared to 1317 in liver and only 256 in heart (Fig. 2B). In addition, Venn comparisons revealed only minimal overlap (∼15%) between liver and spleen, and even less with heart, underscoring a highly tissue-specific proteomic response to mtDNA mutations (Fig. 2B). Notably, the coefficient of variation (CV%, defined as 100 × SD/mean across samples per protein) was comparable across heart, liver, and spleen datasets, showing that the observed difference reflects biological responses rather than technical variability (fig. S2A). The only consistent overlap across all three tissues was a small subset of differentially expressed metabolic enzymes and mitochondrial translation–related proteins (Fig. 2B), consistent with metabolic adaptation and enhanced mitochondrial translation representing conserved core responses triggered by mtDNA mutations in *Polg*^wMut^ mice.

To delineate the nature of these tissue-specific alterations, we next performed an unbiased pathway enrichment analysis on differentially abundant proteins from each organ. In the spleen, the top 20 enriched pathways were dominated by coordinated down-regulation of proteins involved in aerobic respiration, as well as innate and adaptive immune responses, indicating broad functional decline (Fig. 2C). In contrast, proteins involved in DNA replication were up-regulated (Fig. 2C), consistent with the proliferative expansion of hematopoietic precursors during the compensatory extramedullary hematopoiesis in *Polg*^Mut^ mice (*15*, *35*).

The liver demonstrated a more complex pattern of remodeling, with both up- and down-regulation of proteins linked to detoxification, metabolism, and immune regulation. We observed a consistent down-regulation of proteins related to aerobic respiration, indicating impaired OXPHOS (Fig. 2C), consistent with previous reports in *Polg*^Mut^ mice (*15*, *23*). In parallel, we found an innate immune response signature marked by elevated IFN-I–inducible proteins (OAS1A, ISG15, and ZBP1), complement factors (C1QA to C1QC), and antimicrobial effectors (S100A8/9, LTF, and CYBB), alongside down-regulation of adaptive immune proteins/immunoglobulin M–related proteins (IGHM, J-chain) (Fig. 2C). Together, these changes underscore the role of the liver as a central immune-metabolic–responsive organ in the mtDNA mutator context.

In contrast, the heart exhibited selective up-regulation of metabolic enzymes and down-regulation of key components of the complement system (Fig. 2C). Unexpectedly, OXPHOS-related pathways were not among the top-enriched terms in the heart. In addition, OXPHOS subunit abundance in the heart showed greater intersample variability than in the liver or spleen, reflecting the postmitotic state of cardiomyocytes, where uneven clonal expansion of mtDNA mutations leads to marked heterogeneity between cardiomyocytes (*15*). In contrast, continuous cellular turnover in the more proliferative and mitotically active liver and spleen likely mitigates such variability (fig. S3).

### Cardiac metabolic rewiring and aberrant immune response in *Polg*^wMut^ mice

**Fig. 3.**
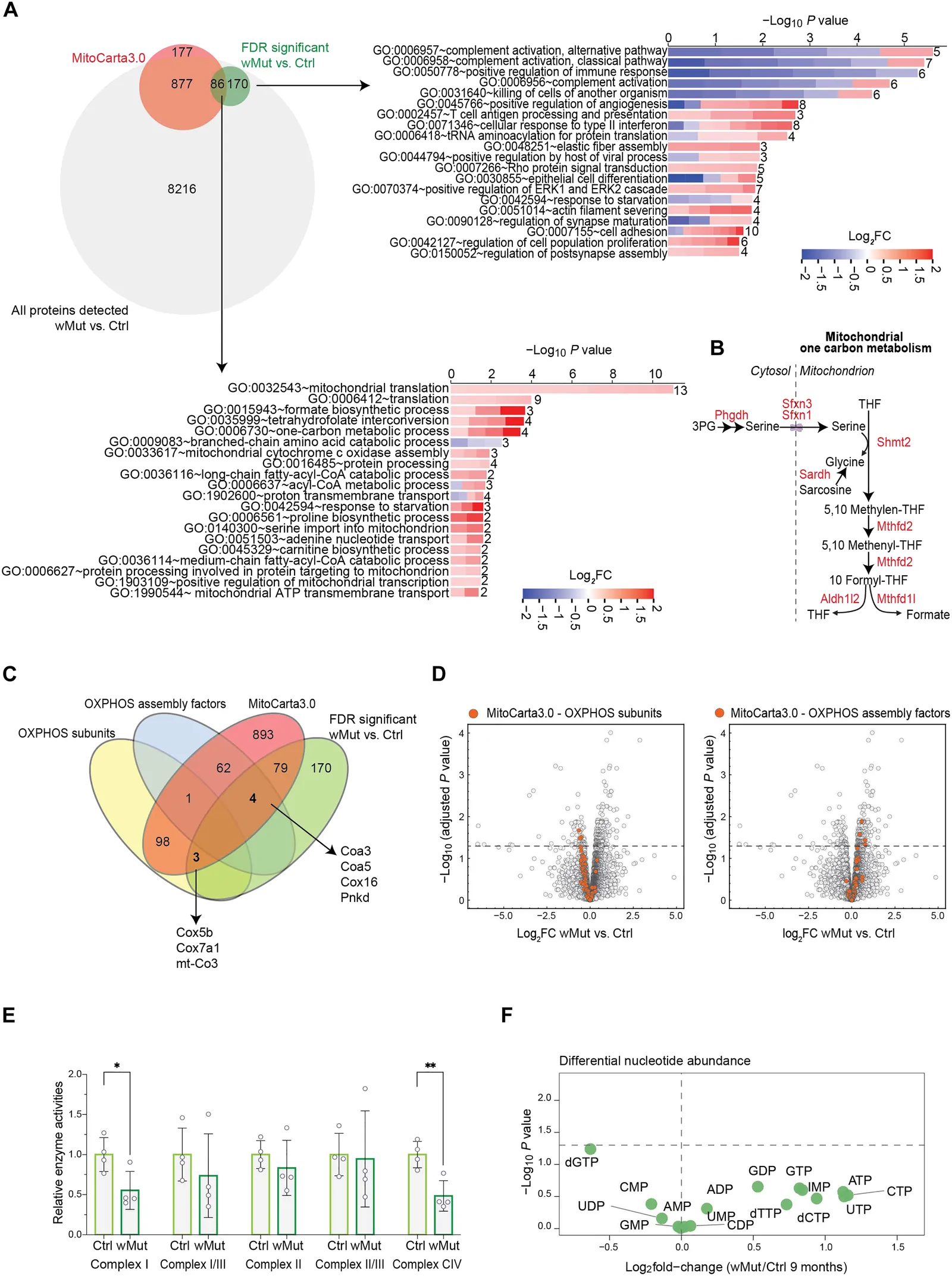
Metabolic rewiring and aberrant immune response in the heart, with minimal effects on core OXPHOS components following whole-body mtDNA mutagenesis. (**A**) Venn diagram showing the overlap of differentially abundant proteins in 9-month-old whole-body *Polg*^wMut^ hearts (green, −log_10_ adjusted *P* value >1.3) with mitochondrial proteins annotated with mouse MitoCarta3.0 (red). The total number of detected proteins is indicated in gray to illustrate the coverage depth of the mitochondrial proteome. Functional enrichment analysis (DAVID) and corresponding cell plots are presented for mouse MitoCarta 3.0 annotated significant proteins (bottom) and non-MitoCarta 3.0 differentially abundant proteins (right). (**B**) Schematic overview of up-regulated enzymes (red) in *Polg*^wMut^ hearts involved in mitochondrial one-carbon metabolism. (**C**) Venn diagram illustrating the proportions and overlaps of OXPHOS subunits and assembly factors with mouse MitoCarta3.0 annotated proteins and the differentially abundant proteins (−log_10_ adjusted *P* value >1.3) in the 9-month-old whole-body *Polg*^wMut^ hearts. (**D**) Volcano plot of cardiac proteome showing OXPHOS structural subunits and OXPHOS assembly factors in 9-month-old whole-body *Polg*^wMut^ hearts (annotated in orange). (**E**) Relative respiratory chain enzyme activities of cardiac mitochondria of 9-month-old control (Ctrl, light green) and whole-body *Polg*^wMut^ mice (wMut, dark green). Data are presented as means ± SD, unpaired two-tailed Welch’s *t* test; *P < 0.05, **P < 0.01. (**F**) Targeted metabolomic analysis of cardiac nucleotide pools in 9-month-old *Polg*^wMut^ hearts. Volcano plots showing nucleotide abundance changes in mutant animals relative to matched controls. Each point represents one nucleotide. The *x* axis shows log_2_ fold change calculated from mean mutant/control abundance, and the *y* axis shows −log_10_ *P* value (Welch’s *t* test).

To analyze how the cardiac proteome, respiratory chain function, and nucleotide levels are altered in response to whole-body mtDNA mutagenesis, we performed additional analyses of the heart from 9-month-old *Polg*^wMut^ mice (Fig. 3, A to F). Approximately 30% of the significantly altered proteins were mitochondrially localized according to the mouse MitoCarta3.0 annotation (Fig. 3A). Pathway enrichment analysis showed up-regulation of mitochondrial translation and one-carbon metabolism, with all key enzymes of the mitochondrial one-carbon pathway increased in abundance (Fig. 3, A and B). In contrast, proteins involved in branched-chain amino acid catabolism were consistently down-regulated, indicating a coordinated metabolic shift (Fig. 3A, bottom). This pattern is consistent with a conserved metabolic response to mitochondrial dysfunction that has been linked to the maintenance of redox homeostasis, antioxidant defense, and nucleotide biosynthesis under bioenergetic stress (*36*–*39*). We therefore investigated whether downstream metabolic consequences could be detected in *Polg*^wMut^ hearts. We performed targeted metabolomic analysis of cardiac nucleotide pools and examined oxidative stress–associated markers. Nucleotide pools were largely unchanged in 9-month-old *Polg*^wMut^ hearts, showing that the observed induction of mitochondrial one-carbon metabolism is not accompanied by major changes in steady-state nucleotide abundance at this stage (Fig. 3F). In contrast, lipid hydroperoxides and protein-bound malondialdehyde (MDA) were increased, consistent with elevated lipid peroxidation, whereas protein carbonylation was unchanged, suggesting no detectable increase in direct protein oxidation (fig. S4, B to D).

Unexpectedly, only a minor fraction of differentially abundant mitochondrial proteins corresponded to OXPHOS subunits or assembly factors (Fig. 3C). However, a consistent trend toward decreased structural subunit and increased assembly factor levels was observed (Fig. 3D). Enzyme activity assays revealed ∼50% reduction in complex I and IV activity (Fig. 3E), showing a functional respiratory chain impairment despite the relatively preserved steady-state protein levels.

Around 70% of differentially abundant proteins were nonmitochondrial and showed notable enrichment of immune-related pathways (Fig. 3A, right). Proteins linked to T cell antigen processing and presentation (RFTN1, UNC93B1, and ICAM1) and cytoskeletal regulation via Rho signaling were up-regulated (Fig. 3A). Notably, components of the complement system were highly overrepresented, with key components such as C3 and C5AR significantly increased, whereas terminal components of the membrane attack complex (C6, C8A/B/G, and C9) were reduced (Fig. 3A), consistent with chronic complement activation and consumption. Together, these findings show that whole-body mtDNA mutations elicit a response in *Polg*^wMut^ hearts characterized by coordinated metabolic adaptation, increased oxidative stress, and an altered immune response.

### Activation of homozygous POLG^D257A^ expression in the heart

**Fig. 4.**
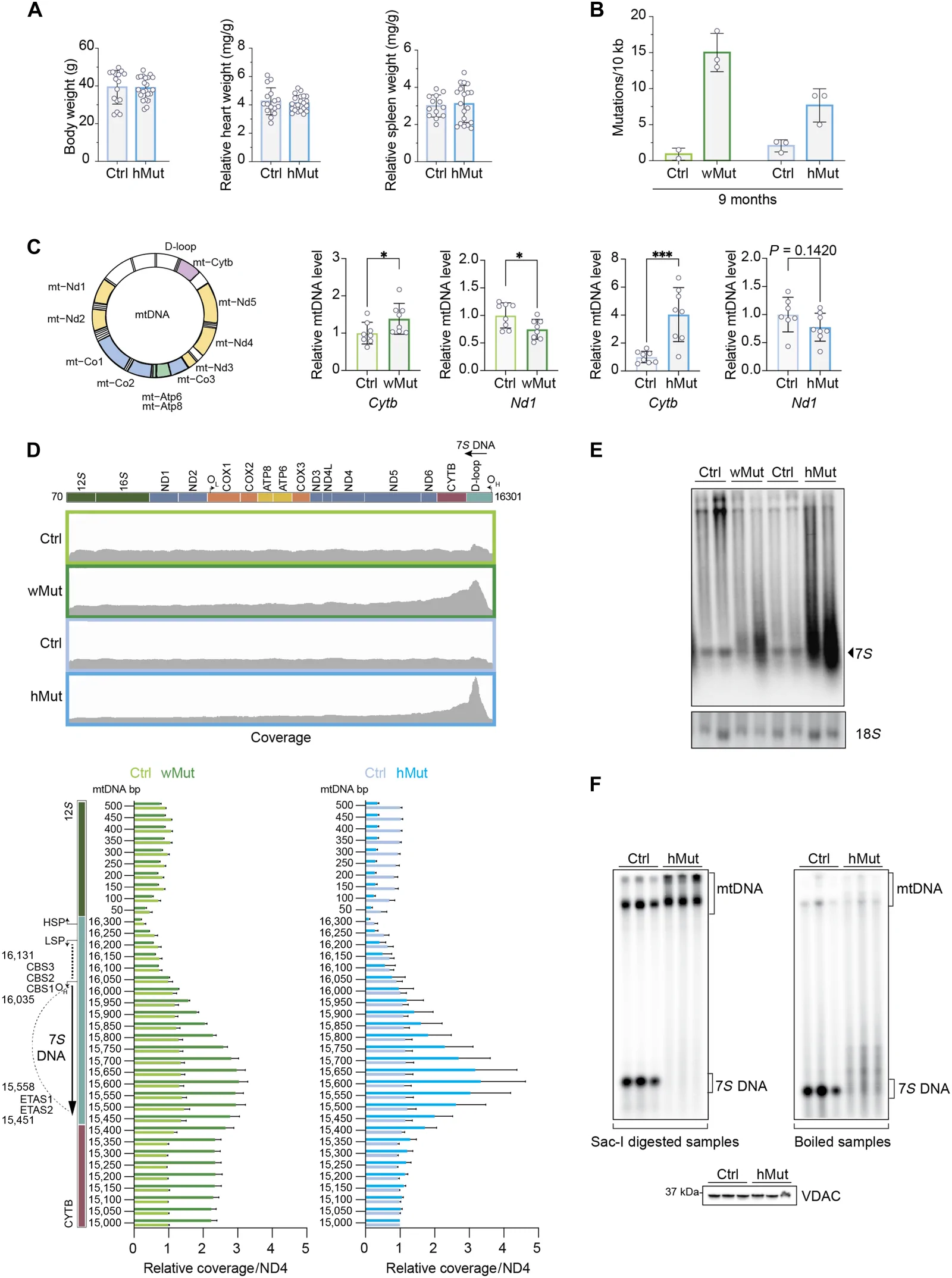
Tissue-specific induction of the POLG^D257A^ expression in heart. (**A**) Body and organ weights normalized to body weight (milligrams per gram) are shown for 9-month-old control (Ctrl) and *Polg*^hMut^ mice (hMut). (**B**) Total number of somatic mtDNA mutations in heart tissue was quantified by PCR amplification, cloning, and sequencing. Mutation frequency is expressed as mutations per 10-kb mtDNA and compared between whole-body (*Polg*^wMut^) and tissue-specific (*Polg*^hMut^) models and their respective controls at 9 months of age. (**C**) Relative mtDNA copy number in 9-month-old hearts of whole-body *Polg*^wMut^ (green) and *Polg*^hMut^ mice (blue) was analyzed by quantitative real-time PCR (qPCR) using *Cytb* and *Nd1* probes normalized to 18*S* nuclear rDNA. Data are presented as means ± SD, unpaired two-tailed Welch’s *t* test; *P < 0.05; ***P < 0.001. (**D**) NGS-based mitochondrial genome coverage from mtDNA isolated from 9-month-old *Polg*^wMut^ and *Polg*^hMut^ hearts. Relative coverage of the D-loop region is plotted in bar graphs (bottom). (**E**) 7*S* detection by Southern blot analysis of DNA isolated from 9-month-old *Polg*^wMut^ and *Polg*^hMut^ hearts. (**F**) In organello mtDNA replication assay in isolated heart mitochondria from 9-month-old control (Ctrl) *Polg*^hMut^ mice (hMut). Left: Samples digested with SacI; right: boiled samples. Bottom panel shows VDAC immunoblot as loading control, with protein aliquots taken before DNA extraction for the in organello assay.

Both systemic and heart-intrinsic effects may shape the heart phenotype in *Polg*^wMut^ mice because of the ubiquitous accumulation of mtDNA mutations in all tissues, including hematopoietic and immune cells. To study the tissue-intrinsic role of mtDNA mutations, we crossed *Polg*^iMut^ mice with Ckmm-cre mice to generate mice with homozygous expression of POLG^D257A^ in the heart (henceforth denoted *Polg*^hMut^ mice; fig. S1B). Unlike the *Polg*^wMut^ mice, the *Polg*^hMut^ mice maintained normal body and organ weights at 9 months of age (Fig. 4A), indicating absence of overt systemic pathology. Quantification of total somatic mtDNA mutations in the hearts of 9-month-old *Polg*^hMut^ mice showed that they had approximately half the mutation load observed in *Polg*^wMut^ mice (Fig. 4B), consistent with the restriction of mutagenesis to cardiomyocytes and delayed onset of mtDNA mutagenesis in mid-gestation in *Polg*^hMut^ mice.

To evaluate how accumulating mtDNA mutations affect genome maintenance, we quantified mtDNA copy number in hearts of *Polg*^wMut^ and *Polg*^hMut^ mice by using quantitative polymerase chain reaction (qPCR) assays of different mtDNA regions. Analysis of mtDNA in heart from *Polg*^wMut^ and *Polg*^hMut^ mice showed elevated levels of the CytB region, whereas the Nd1 region was less abundant (Fig. 4C). We proceeded with next-generation sequencing (NGS) and found a marked enrichment of read coverage across the D-loop extending into the CytB region in both models (Fig. 4D), consistent with accumulation of replication intermediates and duplications in this region, as previously described (*15*, *40*). Southern blot analyses of mtDNA in heart demonstrated elevated levels of 7*S* DNA in both models (Fig. 4E). We also performed in organello assays and found robust de novo mtDNA replication accompanied by very low levels of 7*S* DNA in mitochondria from *Polg*^hMut^ hearts (Fig. 4F). Boiling of samples released a smear of replication intermediates in the 7*S* DNA region (Fig. 4F, right) consistent with the pattern seen on NGS (Fig. 4D) and Southern blots (Fig. 4E). The single-stranded 7*S* DNA represents an abortive mtDNA replication product that is continuously formed and degraded (*41*). Extension of 7*S* DNA strands is believed to be an important regulatory step to achieve genome-length mtDNA replication (*41*). The *MGME1* homozygous knockout mice show a similar increase of 7*S* DNA (*42*, *43*), arguing that impaired mtDNA replication affects the formation and/or usage of 7*S* DNA.

### Cardiomyocyte-intrinsic mtDNA mutations trigger early mitochondrial stress responses

**Fig. 5.**
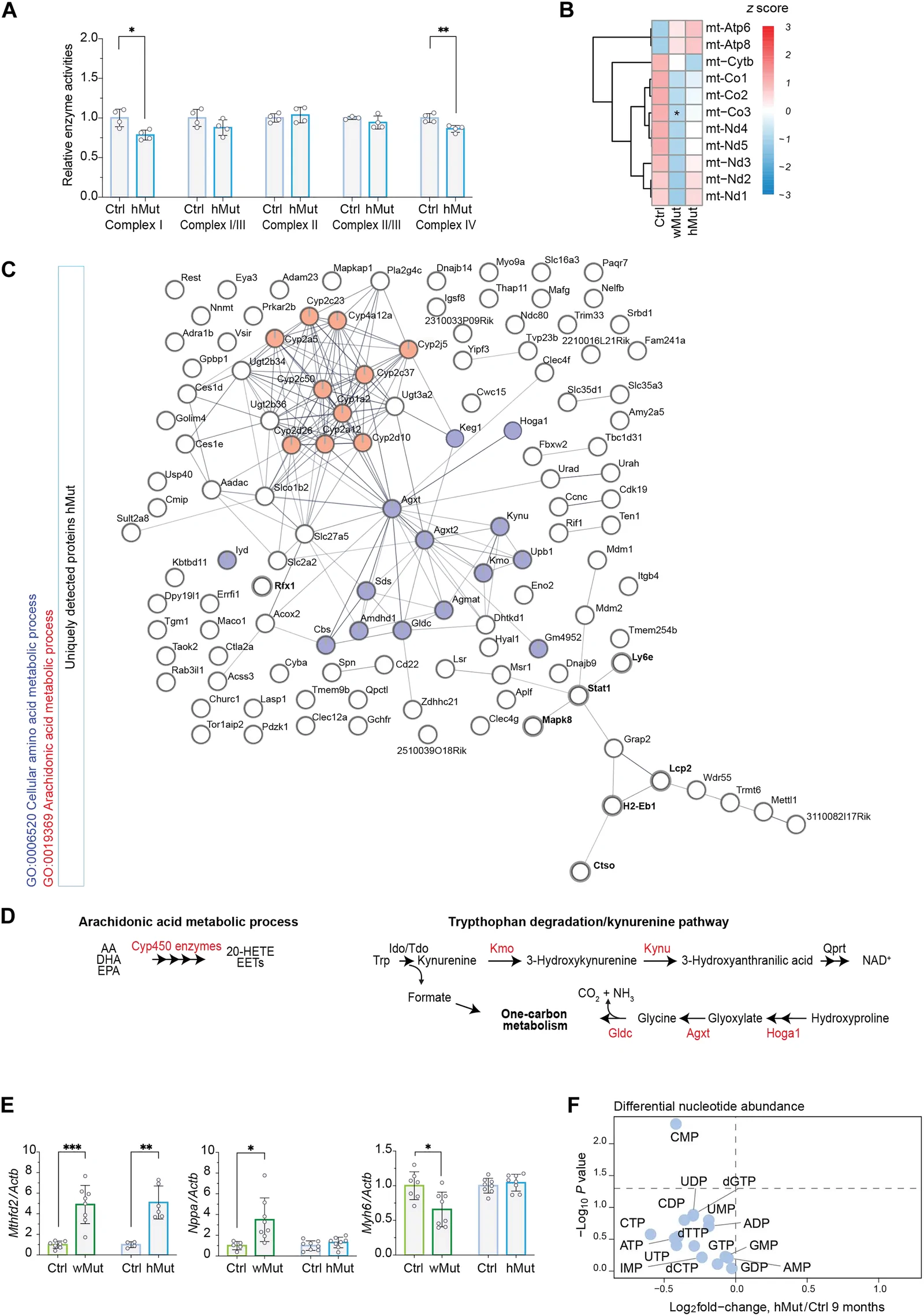
Early cardiac response to accumulated mtDNA mutations in 9-month-old *Polg*^hMut^ mice. (**A**) Relative respiratory chain enzymatic activities of cardiac mitochondria of 9-month-old control (Ctrl) and *Polg*^hMut^ mice (hMut). Data are presented as means ± SD, unpaired two-tailed Welch’s t test; *P < 0.05, **P < 0.01 (**B**) Heatmap showing *z* score–normalized average abundance of mtDNA encoded proteins detected by proteomic analysis in 9-month-old heart tissue from control, *Polg*^wMut^, and *Polg*^hMut^ mice [* adjusted *P* value (FDR) <0.05]. (**C**) Proteins uniquely detected in at least three of five *Polg*^hMut^ hearts were visualized using STRING network analysis. Proteins associated with cellular amino acid metabolic process (GO:0006520) are shown in blue and AA metabolic process proteins (GO:0019369) in red. Proteins discussed in the text (STAT1, MAPK8/JNK1, LY6E, H2-EB1, RFX1, CTSO, and LCP2) are highlighted in bold. (**D**) Schematic overview of biological pathways uniquely induced in 9-month-old *Polg*^hMut^ hearts (red, increased protein abundance). (**E**) Relative transcript levels of *Mthfd2*, *Nppa*, and *Myh6* normalized to *Actb* in 9-month-old *Polg*^wMut^ (green) and *Polg*^hMut^ mice (blue). Data are presented as means ± SD, unpaired two-tailed Welch’s *t* test; *P < 0.05, **P < 0.01, ***P < 0.001. (**F**) Targeted metabolomic analysis of cardiac nucleotide pools in 9-month-old *Polg*^hMut^ hearts. Volcano plots showing nucleotide abundance changes in mutant animals relative to matched controls. Each point represents one nucleotide. The *x* axis shows log_2_ fold change calculated from mean mutant/control abundance, and the *y* axis shows −log_10_ *P* value (Welch’s *t* test).

To determine whether cardiomyocyte-intrinsic mtDNA mutations are sufficient to elicit responses similar to those observed in heart of whole-body mutant *Polg*^wMut^ mice, we first assessed OXPHOS complex activities. Consistent with the approximately 50% lower mtDNA mutation burden in this model (Fig. 4B), complex I and IV enzyme activities were only reduced by ∼25% (Fig. 5A), compared to the ∼50% reduction observed in the heart of *Polg*^wMut^ mice (Fig. 3E).

Quantitative proteomics of hearts from 9-month-old *Polg*^hMut^ mice revealed no FDR-significant (*P* adjusted >0.05) changes in protein abundance. However, mtDNA-encoded proteins showed a modest reduction in mean abundance, mirroring the more pronounced pattern in whole-body mutants (Fig. 5B). When comparing heart, liver, and spleen datasets, mutant hearts stood out by displaying the highest number of uniquely detected proteins, defined as proteins detected in mutants but absent in all corresponding controls, suggesting tissue-specific biological effects (fig. S2B). STRING network analysis of proteins uniquely detected in at least three of five 9-month-old *Polg*^hMut^ hearts, revealed two prominent functional clusters (Fig. 5C) detected at quantitatively robust intensity levels (fig. S2C). We found an enrichment for cytochrome P450 enzymes, which oxidize arachidonic acid (AA), docosahexaenoic acid (DHA), and eicosapentaenoic acid (EPA) into bioactive lipid mediators such as epoxyeicosatrienoic acids and 20-hydroxyeicosatetraenoic acid (*44*, *45*), alongside enzymes involved in amino acid metabolism, particularly tryptophan and hydroxyproline catabolism (Fig. 5, C and D), indicating early metabolic stress signaling. Supporting this observation, transcript levels of the mitochondrial stress–responsive gene *Mthfd2* were similarly elevated in hearts of 9-month-old *Polg*^wMut^ and *Polg*^hMut^ mice. In contrast, classical cardiac stress markers such as *Nppa* and *Myh6* were only altered in *Polg*^wMut^ mice, indicating that pathological remodeling had not yet occurred in the *Polg*^hMut^ hearts (Fig. 5E).

In addition, a third cluster emerged in the STRING network analysis, suggesting a molecular signature of IFN-driven immune response (Fig. 5C). We detected unique abundance of STAT1, MAPK8 (JNK1), and LY6E, that all are IFN-stimulated proteins. Despite the absence of classical immune cell markers, MHC class II component (H2-EB1) and the transcriptional activator RFX1, a key driver of MHC class II gene expression in response to inflammatory stimuli such as IFN-γ, were induced. Furthermore, proteins involved in antigen processing, including the lysosomal protease CTSO were uniquely detected, as well as LCP2, an adaptor protein mediating immune cell signal transduction (Fig. 5C), suggesting activation of immune-related signaling pathways. Together, these findings demonstrate that intrinsic mtDNA mutation accumulation initiates early mitochondrial stress and metabolic adaptation in the heart, accompanied by elevated abundance of immune-related proteins even in the absence of systemic pathology or pronounced morphological cardiac changes.

### Cardiomyocyte-intrinsic mtDNA mutations drive progressive contractile dysfunction

**Fig. 6.**
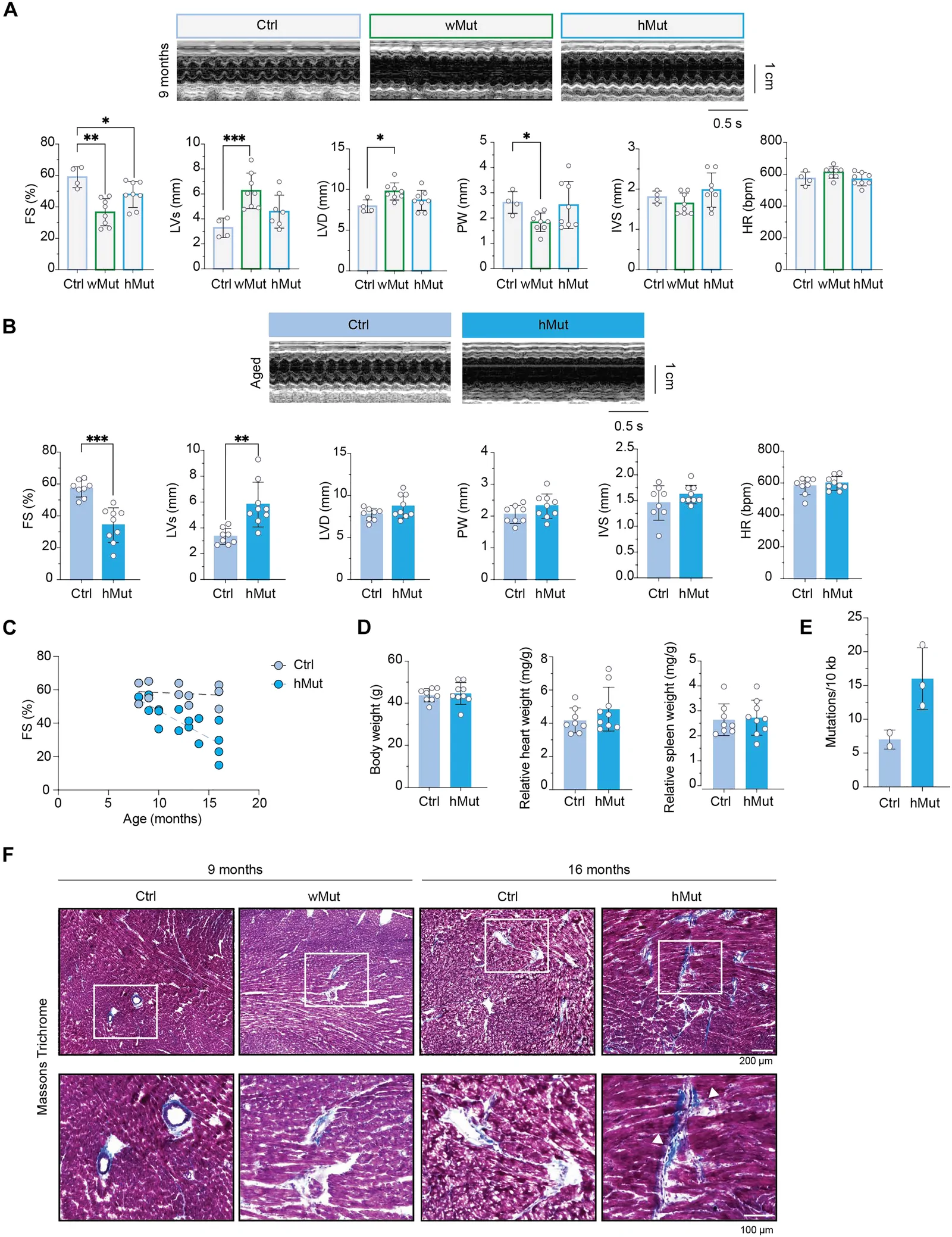
Accumulation of mtDNA mutations impairs cardiac contractility in both *Polg*^wMut^ and *Polg*^hMut^ mice. (**A**) Echocardiographic analysis of 9-month-old control (Ctrl), *Polg*^wMut^ (wMut) and *Polg*^hMut^ (hMut) mice (**B**) Echocardiographic analysis of aged controls and *Polg*^hMut^ mice (12 to 16-months-old)**.** Representative M-mode images and quantification of cardiac functional parameters are shown. FS%; LVs (millimeter), LVD (millimeter); PW (thickness, mm); IVS (millimeter); HR (heart rate, bpm). (**C**) Correlation analysis between FS% and age in *Polg*^hMut^ mice. (**D**) Body and relative organ weight (milligrams per gram) of aged *Polg*^hMut^ mice. (**E**) Total number of somatic mtDNA mutations in 16-month-old *Polg*^hMut^ hearts was determined by PCR, cloning, and sequencing. Data are presented as means ± SD, unpaired two-tailed Welch’s *t* test; *P < 0.05, **P < 0.01, ***P < 0.001. (**F**) Masson’s trichrome staining of cardiac sections from 9-month-old control and *Polg*^wMut^ hearts, and 16-month-old control and *Polg*^hMut^ hearts. Representative lower and higher-magnification images are shown. Blue staining indicates collagen deposition. Arrows indicate fibrotic regions containing multiple nuclei. Scale bars, 200 μm (top) and 100 μm (bottom). The 16-month-old control (Ctrl) and *Polg*^hMut^ Masson’s trichrome images were generated from the same original stained images used to generate the corresponding control and second *Polg*^hMut^ Masson’s trichrome images in fig. S8A.

To assess consequences of mtDNA mutation accumulation on cardiac function, we performed echocardiographic analysis in *Polg*^hMut^ and *Polg*^wMut^ mice. At 9 months, *Polg*^wMut^ mice exhibited pronounced systolic dysfunction, evidenced by an ∼50% reduction in fractional shortening (FS), significant increases in left ventricular end-systolic and end-diastolic diameters (LVs and LVDs), and reduced posterior wall thickness (PW), consistent with impaired contractility associated with dilated cardiomyopathy and pathological cardiac remodeling (Fig. 6A). In contrast, age-matched *Polg*^hMut^ mice showed only an ∼25% reduction in FS without chamber dilation or wall thinning, suggesting isolated early contractile impairment in the absence of structural remodeling (Fig. 6A). In aged *Polg*^hMut^ mice (12 to 16 months), FS declined further, accompanied by LVs expansion (Fig. 6B), reaching values similar to those seen in 9-month-old *Polg*^wMut^ mice (Fig. 6A). Correlation analysis revealed a progressive age-dependent decline in FS in *Polg*^hMut^ mice, whereas it remained stable in aging controls (Fig. 6C). Despite this functional impairment, body, heart, and spleen weights remained unchanged in aged *Polg*^hMut^ mice (Fig. 6D). Additional histological analysis revealed local tissue remodeling in mutant hearts. Masson’s trichrome staining showed focal collagen deposition in both 9-month-old *Polg*^wMut^ and 16-month-old *Polg*^hMut^ hearts, with the most pronounced fibrotic remodeling observed in *Polg*^hMut^ hearts (Fig. 6F and fig. S8A). These findings indicate that progressive contractile dysfunction is accompanied by local fibrotic remodeling. The cardiac functional decline correlated with a progressive increase in the cardiac mtDNA mutation burden, which by 16 months reached levels observed in 9-month-old *Polg*^wMut^ mice (Fig. 6E). These findings demonstrate that accumulation of mtDNA mutations in cardiomyocytes is sufficient to drive progressive cardiac contractile dysfunction and fibrosis, independent of systemic pathology.

### Cardiomyocyte-intrinsic mtDNA mutations trigger metabolic rewiring, immune response, and heterogeneous OXPHOS decline in the aging heart

**Fig. 7.**
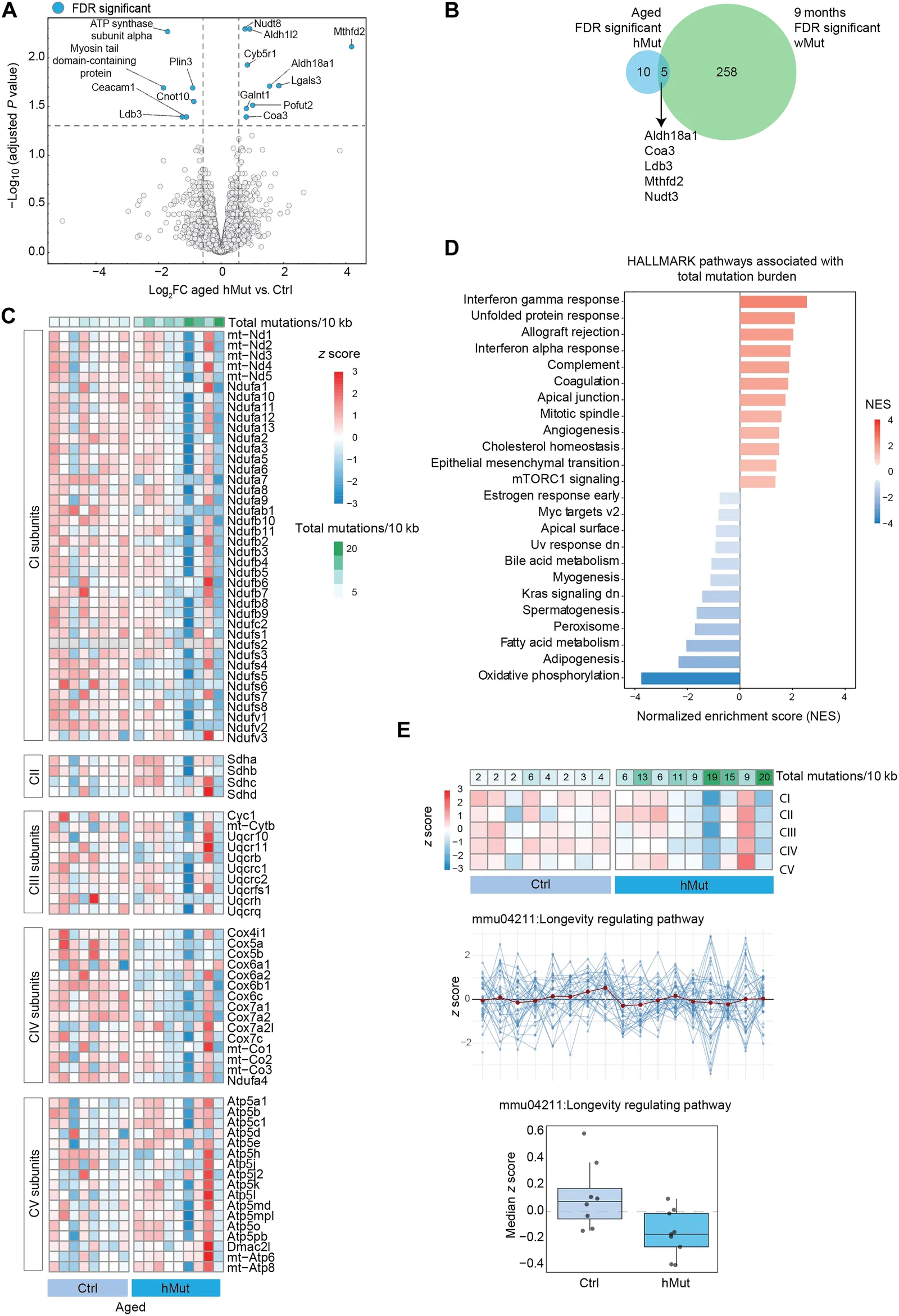
Aged *Polg*^hMut^ hearts show metabolic rewiring, heterogeneous OXPHOS alterations, and signs of cardiac aging. (**A**) Volcano plot visualizing significantly regulated proteins in aged *Polg*^hMut^ hearts (blue = **−**log_10_ adjusted *P* value >1.3). (**B**) Venn diagram comparing proteomic signatures between differentially abundant proteins detected in 9-month-old *Polg*^wMut^ and aged *Polg*^hMut^ mice hearts. (**C**) Heatmap showing *z* score–normalized expression of OXPHOS subunits from proteomic data of aged control (Ctrl) and *Polg*^hMut^ heart samples (hMut) The top annotation bar indicates total cardiac mtDNA mutation burden (total mtDNA mutations/10 kb). (**D**) Gene set enrichment analysis of proteomics data from aged *Polg*^hMut^ hearts. Proteins were ranked according to their Spearman correlation with total mtDNA mutation burden. Pathways shown in red were enriched among proteins that increased with mutation burden, whereas pathways shown in blue were enriched among proteins that decreased with mutation burden. (**E**) Line plots showing *z* scores of genes in the KEGG mmu04211 (Longevity regulating pathway) across aged control (Ctrl) and *Polg*^hMut^ heart samples. Individual gene expression profiles are shown in blue, with the mean *z* score per sample overlaid in red. Bottom, a heatmap displays mean abundances of OXPHOS subunits I to V, with a top annotation panel indicating total mtDNA mutations per 10 kb. Total mtDNA mutation values are shown within each box. Boxplot shows the median *z* score across all genes in this pathway. Jittered points represent individual sample values.

To investigate whether a comparable degree of contractile impairment elicits similar proteomic adaptations in tissue-specific versus whole-body mtDNA mutator mice, we analyzed the cardiac proteome of aged *Polg*^hMut^ mice and compared it to 9-month-old *Polg*^wMut^ mice (Fig. 7, A and B). Although both models exhibit comparable mtDNA mutation burdens and similar degrees of contractile dysfunction, *Polg*^hMut^ hearts showed a markedly reduced number of differentially abundant proteins (Fig. 7, A and B). Direct comparison revealed minimal overlap with the proteomic profile of *Polg*^wMut^ mice (Fig. 7B), highlighting distinct molecular trajectories despite similar physiological outcomes. Notably, when we selectively compared controls only to *Polg*^hMut^ hearts with pronounced OXPHOS subunit depletion, the number of significant hits increased slightly but did not reveal additional regulatory pathways (fig. S5, A and B). Instead, metabolic rewiring remained the dominant signature, with OXPHOS subunit depletion as the only additional feature. The minimal overlap with the *Polg*^wMut^ heart profile in this targeted analysis supports the conclusion that extensive proteomic remodeling in the whole-body model is largely amplified by systemic cues rather than being the consequence of heart-intrinsic mtDNA mutations. Consistent with this interpretation, complementary ultrastructural and oxidative stress marker analysis also differed between the models. Whereas 9-month-old *Polg*^wMut^ hearts showed more pronounced oxidative damage and mitochondrial ultrastructural abnormalities, 16-month-old *Polg*^hMut^ hearts did not show broad oxidative stress damage and mitochondrial organization and cristae morphology were largely preserved in *Polg*^hMut^ hearts, with only occasional focal intramitochondrial regions showing apparent cristae loss or disorganization (fig. S4, B to D and F). Metabolomic analyses further supported this model-specific distinction. Whereas nucleotide pools were largely unchanged in 9-month-old *Polg*^wMut^ hearts (Fig. 3F), *Polg*^hMut^ hearts showed age-dependent coordinated remodeling of nucleotide abundance (Fig. 5F and fig. S4A). However, adenosine triphosphate (ATP) levels were preserved in both models, and adenosine monophosphate–activated protein kinase (AMPK) and ACC phosphorylation was not altered in 16-month-old *Polg*^hMut^ hearts, indicating no energetic crisis, despite reduced OXPHOS subunit abundance (fig. S4E).

Heatmap analysis of OXPHOS subunits revealed markedly greater interindividual variability in subunit abundance in aged *Polg*^hMut^ hearts compared to whole-body mutants. Responses ranged from preserved to severely depleted or up-regulated OXPHOS subunit abundances (Fig. 7C and fig. S3). To determine whether this heterogeneity was related to mtDNA mutation burden within individual animals, we quantified total mtDNA mutation burden in the same aged *Polg*^hMut^ hearts used for proteomic analysis and found that hearts with higher mutation burdens generally exhibited lower OXPHOS subunit abundance (Fig. 7, C and E). In contrast to the whole-body model where early onset ubiquitous mutagenesis results in more uniform mutation propagation, the later onset of POLG^D257A^ expression in *Polg*^hMut^ hearts leads to stochastic somatic mutation accumulation and clonal expansion, causing greater variability in OXPHOS subunit abundance and mtDNA mutation load among individual hearts. Despite this heterogeneity, FDR-corrected significant hits represented the up-regulation of metabolic enzymes (Fig. 7A), mirroring adaptations observed in whole-body mutants. In parallel, the regulation of immune-related proteins emerged as a recurrent feature. The anti-inflammatory mediator CEACAM1 was down-regulated, whereas LGALS3, a proinflammatory mediator and marker of cardiac fibrosis, was up-regulated (Fig. 7A). STRING network analysis of uniquely detected proteins in the heart sample with the most pronounced OXPHOS subunit depletion identified proteins involved in IFN signaling, innate immune sensing, antigen presentation, and immune cell recruitment (fig. S2E), suggesting a direct link between the severity of mitochondrial respiratory impairment and activation of immune responses. These proteins were detected at quantitatively robust intensity levels, as demonstrated by an intensity versus protein rank plot, confirming that their detection represents a biologically meaningful signal rather than low-abundance noise (fig. S2D). To further dissect this relationship, we generated heatmaps and line plots depicting the abundance of IFN-stimulated proteins and proteins annotated to GO:0019886 (antigen presentation via MHC class II) across individual samples, revealing variable but coordinated abundance patterns (fig. S6, A and B). Corresponding heatmaps of OXPHOS subunit (I-V) abundance demonstrated that samples with decreased OXPHOS protein levels exhibited a reciprocal increase in immune pathway proteins, suggesting a correlation between mitochondrial respiratory dysfunction and immune response (fig. S6, A and B). To quantify this relationship across individual aged *Polg*^hMut^ hearts, we ranked all detected proteins by their Spearman correlation with total mtDNA mutation burden and performed gene set enrichment analysis. Increasing mutation burden was associated with negative enrichment of OXPHOS, whereas IFN responses were positively enriched (Fig. 7D). To validate the immune component of this response at the transcript level, we further analyzed bulk RNA sequencing (RNA-seq) data from aged *Polg*^hMut^ hearts. RNA-seq showed an even more pronounced mutation burden–associated immune signature, including positive enrichment of IFN, inflammatory, complement, and apoptosis-associated pathways, together with negative enrichment of OXPHOS (fig. S7A). Heatmap analysis of canonical IFN-stimulated and antigen-presentation genes confirmed coordinated induction of these transcripts in high-mutant *Polg*^hMut^ hearts (fig. S7B). Consistent with this pathway-level enrichment, heatmap analysis of genes annotated to apoptotic process (GO:0006915) showed increased expression of apoptosis-associated transcripts in *Polg*^hMut^ hearts, supporting activation of cellular stress and injury-related programs in the mutant hearts (fig. S7C).

These findings were supported by complementary histological and cellular analyses. Cytochrome c oxidase/succinate dehydrogenase (COX/SDH) staining of three individual 16-month-old *Polg*^hMut^ hearts revealed variable degrees of mosaic complex IV (COX)–deficient and complex II (SDH)–positive cardiomyocytes, demonstrating heterogeneous OXPHOS deficiency at the tissue level (fig. S8A, top). Analysis of consecutive sections by Masson’s trichrome staining showed that collagen deposition increased with more pronounced SDH-positive cardiomyocyte accumulation, supporting an association between local OXPHOS deficiency and fibrotic remodeling (fig. S8A, bottom). Wheat germ agglutinin (WGA) staining, which outlines cardiomyocyte membranes, revealed more irregular cardiomyocyte membranes in 16-month-old *Polg*^hMut^ hearts, and increased periostin signal supported fibroblast-associated extracellular matrix remodeling (fig. S8B). Transmission electron microscopy confirmed collagen deposition and the presence of immune cells within the interstitial space at higher resolution (fig. S8C). Last, flow cytometry showed increased CD45^+^ immune-cell infiltration in aged *Polg*^hMut^ hearts and revealed changes across several immune-cell populations, with dendritic cells showing a particularly prominent increase (fig. S9, C and D). Tetramethylrhodamine methyl (TMRM) staining of isolated cardiomyocytes provided an independent cellular readout of mitochondrial dysfunction, and animal-matched analysis showed that reduced cardiomyocyte mitochondrial membrane potential was associated with increased cardiac CD45^+^ immune-cell abundance (fig. S9, A and B). Despite prominent local cardiac immune cell infiltration, serum multiplex cytokine profiling did not reveal broad systemic cytokine elevation. Instead, selected circulating factors, including GDF-15 and soluble ICAM-1, were increased, consistent with mitochondrial stress responses and leukocyte-endothelial activation (fig. S9E). Together, these findings support a relationship between cardiomyocyte OXPHOS dysfunction, local immune-cell infiltration, and fibrotic tissue remodeling in *Polg*^hMut^ hearts.

Proteins associated with the KEGG longevity-regulating pathway (mmu04211) were uniformly down-regulated across all aged *Polg*^hMut^ hearts, regardless of OXPHOS subunit abundance (Fig. 7D and fig. S6C). Together, these findings demonstrate that mtDNA mutation accumulation in cardiomyocytes autonomously triggers metabolic reprogramming and immune responses, independent of systemic pathology. The consistent down-regulation of longevity-associated signaling pathways, irrespective of OXPHOS severity, points to a direct connection between mtDNA mutations and activation of aging-related programs.

## DISCUSSION

The *Polg*^Mut^ mouse model induces ubiquitous mtDNA mutation accumulation, making it impossible to separate direct tissue-specific effects from indirect systemic consequences, and this limits our ability to fully understand how mtDNA mutations contribute to organ-specific pathologies and aging. To overcome this limitation, we developed and applied a tissue-specific *Polg*^hMut^ model to study the direct effects of mtDNA mutations, allowing us to understand how cardiomyocyte-intrinsic mtDNA mutations provoke cardiac remodeling and aging.

Our data demonstrate that mtDNA mutations in cardiomyocytes are sufficient to cause progressive contractile dysfunction even in the absence of systemic pathology. The functional decline correlates with increased mtDNA mutation burden and reduced OXPHOS complex activity and occurs before pronounced reductions in OXPHOS subunit abundance. This indicates that reduced OXPHOS complex activity precedes proteomic remodeling and is likely caused by the incorporation of dysfunctional mtDNA–encoded OXPHOS subunits that impair respiratory chain efficiency and contractile function.

As mtDNA mutations accumulated in *Polg*^hMut^ hearts, proteomic profiling revealed an early coordinated metabolic adaptation. One of the most prominent changes was the up-regulation of cytochrome P450 enzymes, which convert AA, DHA, and EPA into bioactive lipid mediators that modulate vascular tone, inflammation, and cell survival, and are implicated in both protective and detrimental outcomes in human heart failure and experimental cardiomyopathy models (*46*–*51*). In parallel, we observed increased abundance of enzymes in the kynurenine pathway and hydroxyproline degradation consistent with enhanced tryptophan catabolism, NAD^+^ biosynthesis, and glycine/formate production for one-carbon metabolism. Elevated serum kynurenine levels are a recognized feature of cardiovascular pathology, including atherosclerosis and myocardial infarction (*52*). Notably, the rate-limiting enzyme of kynurenine production, IDO1, is described to be strongly induced by IFN signaling (*53*, *54*), suggesting that this metabolic reprogramming may be an early manifestation of an immune response triggered by cardiomyocytes harboring mtDNA mutations.

Consistent with this finding, the cardiac proteome exhibits a distinct IFN-driven immune signature already at early stages of mtDNA mutation accumulation. In young *Polg*^hMut^ hearts, we uniquely detected IFN-stimulated proteins alongside induction of MHC class II components and the transcriptional regulator RFX1, a mediator of IFN-γ–dependent MHC class II expression (*55*, *56*). However, because these analyses were performed on whole-heart homogenates, the cellular source of these immune signatures cannot be resolved. The detected proteins may reflect cardiomyocyte-intrinsic stress responses, activation of endothelial or stromal cells, subtle immune-cell infiltration, or a combination of these compartments.

As mtDNA mutations accumulate, the initial local mitochondrial stress response progresses into a broader immunological activation. In aged *Polg*^hMut^ hearts that have pronounced OXPHOS subunit depletion, this immune response is markedly enhanced and is characterized by elevated levels of IFN-stimulated (OAS1A and IRF5), antigen presentation (H2-DM), and costimulatory (CD5 and CD86) proteins and transcripts, alongside increased levels of myeloid and lymphoid markers, which indicates innate sensing and engagement of adaptive immunity in the heart. Together, these findings support a model in which mtDNA mutation–induced respiratory chain failure initiates a localized stress response that ultimately drives inflammation and immune cell infiltration.

Mechanistically, the induction of IFN-stimulated and antigen-presentation programs is compatible with innate immune activation downstream of mitochondrial stress, and prior work in *Polg*^Mut^ mice has implicated mitochondrial nucleic acid sensing and IFN-I signaling (*23*). However, ISG expression was most evident in animals with higher mtDNA mutation burden, indicating that innate immune activation emerges in the context of high mtDNA mutation load rather than as a uniform early response across all mutant hearts. The RNA-seq dataset also revealed uniform induction of apoptosis- and stress-related pathways, raising the possibility that the IFN/immune signature may arise, at least in part, as a secondary response to cardiomyocyte stress, injury, or damage-associated signaling.

A notable finding of our study is the strong correlation between mtDNA mutation burden, the severity of OXPHOS subunit depletion, and the activation level of immune and antigen presentation pathways. Hearts exhibiting the highest mtDNA mutation load and the most pronounced loss of respiratory chain components displayed the highest induction of IFN-stimulated proteins, MHC molecules, and innate immune signaling mediators. This supports a model where progressive mitochondrial dysfunction not only impairs contractile function but also actively licenses and escalates immune responses within the heart. Intriguingly, despite this tight coupling between mtDNA mutation load and immune activation, longevity-associated signaling pathways (mmu04211: Longevity regulating pathway) are consistently down-regulated in *Polg*^hMut^ hearts, independent of the degree of OXPHOS depletion or immune pathway engagement, which argue that mtDNA mutations may drive intrinsic tissue aging via mechanisms distinct from both OXPHOS dysfunction and inflammation, suggesting the involvement of alternative, yet unidentified pathways underlying tissue aging in *Polg*^hMut^ hearts.

In summary, the *Polg*^hMut^ model we present here delineates a potential sequence by which cardiomyocyte-intrinsic mtDNA mutations drive cardiac dysfunction. We demonstrate that the decline in contractile function is primarily caused by mosaic OXPHOS dysfunction, which subsequently triggers a robust immune response marked by immune cell infiltration, which alters the cardiac microenvironment and promotes fibrosis. This model isolates the direct, cell-autonomous consequences of mtDNA mutation accumulation from the systemic effects observed in whole-body models, where cardiac pathology and proteomic remodeling are exacerbated by systemic cues. The independent suppression of longevity pathways suggests additional, distinct mtDNA mutation–induced aging mechanisms yet to be elucidated.

## MATERIALS AND METHODS

### Generation of the inducible *Polg*^*i*Mut^ mtDNA mutator mouse model

The inducible *Polg*^iMut^ mtDNA mutator mouse model was generated with Taconic Biosciences to allow spatial and temporal control of mutant POLG^D257A^ expression [Asp257→Ala (D257A)]. The targeting strategy was based on the *Polg* transcript (NCBI NM_017462.2). The inducible *Polg*^iMut^ allele was generated by targeted insertion of a loxP-flanked cassette comprising fused exons 3 to 23 (cDNA with 3′ untranslated region and an hGH polyadenylation signal) into intron 2 of the endogenous *Polg* locus. Downstream of this cassette, the D257A mutation has been introduced into exon 3. Positive selection markers have been flanked by F3 (puromycin resistance downstream of the proximal loxP site, PuroR) and FRT (neomycin resistance upstream of the distal loxP site, NeoR) sites. The targeting vector was constructed using bacterial artificial chromosome (BAC) clones from the C57BL/6J RPCI-23 BAC library. The linearized construct was electroporated into C57BL/6N Tac embryonic stem cells (Taconic Biosciences). Homologous recombinant clones were selected using double-positive selection for both PuroR and NeoR, and negative selection against thymidine kinase. In vivo Flp recombinase–mediated excision of selection cassettes generated the conditional knock-in *Polg*^iMut^ allele, which expresses the wild-type *Polg* exons 3 to 23. The presence of an hGH polyadenylation signal (hGHpA) downstream of the wild-type cassette prevents transcription of the downstream mutant exon 3. In the absence of cre recombinase, the wild-type Polg cDNA is expressed. Upon cre-mediated recombination, the loxP-flanked wild-type cassette is excised, allowing expression of a downstream exon 3 harboring the proofreading-deficient D257A mutation (GAC➔GCC).

### Animal work

To generate the whole-body mtDNA mutator mouse, *Polg*^+/iMut^ mice were first crossed with +/β-actin–cre transgenic mice (*57*) to induce ubiquitous cre-mediated excision of the loxP-flanked wild-type *Polg* cassette. The recombined *Polg*^iMut^ (*Polg*^rMut^) allele was transmitted through the germline independently of cre-recombinase expression. To remove the cre transgene, *Polg*^+/rMut^; +/β-actin–cre mice were crossed with C57BL/6N and cre-negative offspring were intercrossed to generate homozygous *Polg*^rMut/rMut^ mice (denoted *Polg*^wMut^ mice) and littermate controls. To avoid maternal transmission of preexisting mtDNA mutations, males were bred with C57BL/6N females for colony maintenance. Classical mtDNA mutator mice [here denoted *Polg*^Mut^ and a.k.a. *Polg*^D257A/D257A^ or B6-*Polg*^tm1.1(Lrsn)^ mice] were bred and maintained under the same conditions and used as a reference for validating the inducible model. For tissue-specific induction, *Polg*^+/iMut^ mice were crossed with +/Ckmm-cre transgenic animals (*58*). The resulting offspring were then intercrossed to generate homozygous knock-in mice with POLG^D257A^ expression in heart denoted *Polg*^hMut^ mice (genotype *Polg*^iMut/iMut^; +/Ckmm-cre). Tissues (heart, liver, and spleen) from *Polg*^wMut^ mice were collected at 9 months of age, while hearts from *Polg*^hMut^ mice were either collected at 9 months or from aged animals (12 to 16 months). All animals were housed in individually ventilated cages under a 12-hour light/dark cycle. Mice had ad libitum access to standard chow and water. All procedures were performed in accordance with national and European legislation and FELASA guidelines and were approved by the Stockholm Animal Welfare Ethics Committee under ethical permit number 18936-2022.

### Transthoracic echocardiography

Cardiac function was assessed noninvasively using transthoracic echocardiography with a high-resolution imaging system (Philips HDI 5000) equipped with a CL-15 7-MHz linear-array transducer, as previously described (*59*–*62*). Briefly, control, *Polg*^wMut^, and *Polg*^hMut^ were analyzed at two time points (8 to 10 months and 12 to 16 months), anesthetized with 1 to 2% isoflurane, and placed on a heated platform maintained at 37°C. Thoracic hair was removed using depilatory cream to ensure optimal image quality. Following image acquisition, the mice were kept on the heating pad without anesthesia until full recovery. M-mode recordings were obtained in the parasternal short-axis view. LV contractility was quantified as FS%, calculated using the formula: FS% = (LVd − LVs) / LVd × 100, where LVd and LVs represent the LV internal diameters during diastole and systole, respectively. FS% values from control and tissue-specific mutator mice across different ages were used to generate an FS% versus age correlation curve. Additional parameters, including heart rate, PW thickness, and interventricular septum thickness, were measured.

### Peptide preparation for MS-based proteomics

Approximately 1 mm^3^ of dissected, frozen tissue was transferred into individual wells of a BeatBox Tissue Kit 96x (PreOmics) and overlaid with 80 μl of LYSE buffer (PreOmics). Samples were homogenized in the BeatBox for 10 min at the standard setting, heated at 95°C for 10 min with shaking at 1000 rpm for simultaneous reduction and alkylation, and then subjected to focused ultrasonication on a Covaris instrument at the manufacturer’s standard settings. Protein concentration was determined by BCA assay. For each sample, 10 μg of protein was diluted in 50 mM tris-HCl (pH 8.5) and digested with a trypsin/LysC mix at an enzyme-to-protein ratio of 1:50 (w/w) overnight at 37°C with mild shaking (500 rpm). Digestion was stopped by acidification to a final concentration of 1% formic acid (FA). Acidified peptides were then diluted with 3 to 4 volumes of 1% TFA in isopropanol and loaded onto StageTips packed with two SDB-RPS (Empore) disks, washed once with 1% trifluoroacetic acid (TFA) in isopropanol and once with 0.2% TFA/2% acetonitrile in water, and eluted with 60 μl of 1.25% ammonium hydroxide in 80% acetonitrile. Eluates were dried by vacuum centrifugation and reconstituted in 0.1% FA (EvoA). Peptide concentrations were measured by Nanodrop. For liquid chromatography–mass spectrometry (LC-MS) analysis, 500 ng of peptides per sample was loaded onto Evotip Pure tips (Evosep). All loading steps were performed with 50-μl volumes and centrifugation at 700*g*. Briefly, Evotips were rinsed with EvoB (0.1% FA in acetonitrile), conditioned by soaking in 1-propanol for 3 min until uniformly pale white, and equilibrated with EvoA [0.1% FA in MS-grade water]. The 500-ng peptide samples were loaded in EvoA, washed once with EvoA, and overlaid with EvoA to keep the tips wet. Loaded Evotips were stored submerged in EvoA at 4°C until LC–tandem MS (MS/MS) analysis.

### Quantitative MS

Samples were analyzed using an Evosep One LC system (Evosep) coupled to a timsTOF HT mass spectrometer (Bruker Daltonics; TimsControl/acquisition software 5.0.9). Data acquisition was performed with the 30 samples–per-day method, using an Evosep Performance column (15 cm by 150 μm inside diameter, EV1137, Evosep) maintained at 40°C and interfaced via a nanoelectrospray ion source (Bruker Daltonics) with a 10-μm emitter (ZDV Sprayer 10, Bruker Daltonics). The mobile phases consisted of 0.1% FA in LC-MS–grade water (buffer A) and 99.9% acetonitrile/0.1% FA (buffer B). MS was performed using a dia-PASEF method optimized with py_diaid, comprising 20 dia-PASEF scans, each divided into two ion mobility windows (40 isolation windows in total) with variable isolation widths (11 to 94 Th), covering a mass/charge ratio range of 350 to 1200 and an ion mobility range of 0.70 to 1.30 V s cm^−2^. The instrument was operated with an accumulation and ramp time of 100 ms, a capillary voltage of 1750 V, and collision energy ramped linearly from 20 eV at 1/K0 = 0.6 V s cm^−2^ to 59 eV at 1/K0 = 1.6 V s cm^−2^. Data processing was carried out in DIA-NN (v1.9.1) (*63*) using an in silico–predicted spectral library for the first run, which was then applied to a second search. A FASTA database without isoforms was used. DIA-NN parameters included MS1/MS2 mass accuracy of 15 parts per million, a scan window size of 7, fixed carbamidomethylation, and allowance for one missed cleavage.

### Differential protein abundance analysis

Differential protein abundance was analyzed using the limma package in R Studio (v2024.04.2 + 764). *P* values were adjusted for multiple testing with the Benjamini-Hochberg method. Proteins with an adjusted *P* value <0.05 were considered significant. PCA was performed using the PCAtools package. To identify pathways associated with mtDNA mutation burden, proteins detected in proteomics datasets were ranked according to their Spearman correlation coefficient with total mtDNA mutation burden across individual *Polg*^hMut^ hearts. Gene set enrichment analysis was then performed using the ranked correlation coefficients as input. Enrichment was assessed against MSigDB HALLMARK, Gene Ontology Biological Process, and KEGG pathway collections. Positive normalized enrichment scores (NESs) indicate pathways enriched among genes or proteins positively associated with total mtDNA mutation burden, whereas negative NES values indicate pathways enriched among genes or proteins negatively associated with mutation burden.

### Data visualization

Volcano plots were generated using Instant Clue software (v0.10.10, University of Cologne) (*64*). Venn diagrams were created within the Flaski platform (v3.20.0; https://flaski.age.mpg.de) (*65*), developed by the MPI-AGE Bioinformatics Core Facility. Cell plots were produced with the Flaski application, using the top 20 enriched pathways identified by DAVID functional annotation. Protein-protein interaction networks were generated using the STRING database (https://string-db.org). Heatmaps, boxplots, line plots, and protein rank plots were produced in R Studio (v2024.04.2 + 764) using the tidyverse, pheatmap, ggplot2, dplyr, and ggrepel packages.

### Mitochondrial isolation

Cardiac mitochondria were isolated by differential centrifugation. All steps were performed at 4°C using ice-cold reagents and prechilled equipment. Mice were euthanized by CO_2_, and hearts were collected. Tissues were minced into small pieces and transferred to mitochondrial isolation buffer [MIB; 225 mM sucrose, 20 mM tris-HCl (pH 7.2), and 1 mM EDTA] supplemented with 0.25% trypsin. Samples were gently inverted and incubated for 10 min at 4°C. Following enzymatic digestion, the samples were transferred to glass homogenizers containing MIB supplemented with trypsin inhibitor (0.4 mg/ml). Homogenization was carried out using a loose-fit pestle with 40 strokes at 450 rpm. The homogenate was centrifuged at 1000*g* for 10 min to remove nuclei and cellular debris. Supernatant was collected and centrifuged at 10,000*g* for 10 min to pellet mitochondria. The mitochondrial pellet was resuspended in MIB containing 0.2% bovine serum albumin (BSA) and centrifuged again at 10,000*g* for 10 min. The final pellet was washed once in MIB without BSA and used for downstream applications including enzymatic activity assays or DNA isolation.

### Analysis of mtDNA mutations

Total DNA was extracted from heart tissue samples. The mtDNA mutation load was determined by PCR, cloning, and sequencing as described elsewhere (*15*, *66*). Mouse mtDNA–specific primers, 5′-AGTATAGTAATGCCTGCGGC-3′ and 5′CCTACCCCTAGCCCCCC-3′, were used to amplify a 1-kb region encompassing part of the Nd2 gene, the genes for the Trp, Ala, Asn, Cys, and Tyr transfer RNAs, and a portion of the Co1 gene (nucleotide pair 4920–5953) of mouse mtDNA (GenBank NC_005089.1).

### DNA extraction and mtDNA quantification by qPCR

DNA was extracted from tissue or isolated mitochondria using a phenol/chloroform-based method. Briefly, samples were incubated with 500 μl of lysis buffer [0.5% SDS, 100 mM NaCl, 50 mM tris-HCl (pH 8.0), and 2.5 mM EDTA] and 20 μl of Proteinase K (20 mg/ml). Tissue samples were lysed overnight at 55°C, while isolated mitochondria were lysed for 1 hour. Complete lysis was visually confirmed before proceeding. Subsequently, phenol:chloroform:isoamyl alcohol (Sigma-Aldrich) was added, mixed, and centrifuged at maximum speed for 30 min. The aqueous phase was carefully transferred to a new tube, and an equal volume chloroform was added, vortexed, and centrifuged to remove residual phenol. DNA was precipitated by adding 80 μl of 3 M sodium acetate (pH 5.2) and 1 ml of 99.5% ethanol and then incubated at −80°C for 2 to 3 hours. After centrifugation at 13,000*g* for 30 min, the DNA pellet was washed with 70% ethanol, centrifuged again for 10 min, air-dried, and resuspended in nuclease-free water. DNA concentration was measured using a Nanodrop spectrophotometer.

Extracted DNA from heart tissue was used for mtDNA copy number analysis. qPCR was performed in triplicate using 5 ng of DNA per reaction. Reactions used TaqMan Universal PCR Master Mix II with uracil-*N*-glycosylase (Applied Biosystems) and gene-specific TaqMan probes, run on a Quant Studio 6 Flex system. Mitochondrial targets Nd1 (mm04225274) and Cytb (mm04225271) were quantified and normalized to the nuclear 18*S* rDNA (mm03928990) gene.

### Next-generation sequencing

Isolated mitochondria were treated with deoxyribonuclease (0.03 mg/ml) and ribonuclease (RNase; 0.02 mg/ml) for 1 hour at 37°C to digest nuclear contaminants before proceeding with the phenol-chloroform DNA extraction. DNA was sequenced at Novogene GmbH (Germany). For each sample, 200 ng of high-quality DNA was used as input for library preparation using the NEBNext Ultra DNA Library Prep Kit for Illumina (NEB, USA, catalog no. E7370L), following the manufacturer’s protocol. Briefly, DNA was fragmented to an average size of ∼350 base pairs (bp) by sonication, followed by end-repair, A-tailing, and ligation of Illumina-compatible adapters. The ligated fragments were PCR-amplified and purified using the AMPure XP system (Beckman Coulter, USA). Library quality was assessed using the Agilent 5400 system and quantified by qPCR. Libraries passing quality control were pooled equimolarly and sequenced on an Illumina platform using paired-end 150 bp (PE150) reads. Sequencing reads were mapped to the mouse mitochondrial reference genome. On average, 700× to 7922× genome coverage was generated per sample. Average coverage data were plotted in mtDNA region between 500 and 15,000 bp normalized to ND4.

### Southern blot - 7*S* detection

DNA was extracted from isolated mitochondria using standard phenol-chloroform extraction, and DNA concentration was quantified using a Nanodrop spectrophotometer. For 7*S* DNA detection, 2 μg of DNA was digested with SacI-HF (New England Biolabs) that does not cut within the D-loop region. Following digestion, the samples were heated at 93°C for 5 min to release 7*S* DNA strand and then immediately cooled on ice. Loading dye was added, and samples were resolved on a 0.8% agarose gel containing ethidium bromide in 0.5× TBE buffer. Electrophoresis was performed for ∼15 hours at 30 V to enable optimal resolution of small DNA fragments. After electrophoresis, the gel was sequentially treated with 0.2 M HCl for 10 min, followed by denaturation solution for 20 min (1.5 M NaCl and 0.5 M NaOH), neutralization solution for 20 min (1.5 M NaCl and 1 M tris), and finally 20 min in 20× SSC (3.0 M NaCl, 0.3 M sodium citrate), with water washes (5 min) between each step. DNA was transferred onto Hybond-N+ nylon membranes (Cytiva Amersham) using capillary blotting in 20× SSC buffer overnight. Membranes were rinsed in 2× SSC, air-dried, and ultraviolet (UV) cross-linked at 254 nm (shortwave) using a UVC 500 UV cross-linker (Amersham Biosciences). 7*S* DNA probe corresponding to the full mouse D-loop region (mtDNA positions 15.423 to 16.299) or 18*S* rDNA probe (loading control) was labeled with α-[32P]–deoxycytidine triphosphate (dCTP) using the Prime-It II Random Primer Labeling Kit (Agilent). Hybridization was carried out at 55°C with the α-[32P]-dCTP–labeled double-stranded DNA probe. Membranes were exposed to phosphorimager screens (Typhoon FLA 7000 - GE Healthcare).

### In organello mtDNA replication assay

In organello replication of mtDNA was performed according to previously established protocols (*67*). Briefly, 1 mg of freshly isolated mitochondria were pelleted at 8000*g* for 4 min at 4°C, washed twice in ice-cold incubation buffer [25 mM sucrose, 75 mM sorbitol, 10 mM K_2_HPO_4_, 100 mM KCl, 0.05 mM EDTA, 5 mM MgCl_2_, 10 mM tris-HCl (pH 7.4), 10 mM glutamate, 2.5 mM malate, BSA (1 mg/ml), and 1 mM adenosine diphosphate], and resuspended in 500 μl of the same buffer prewarmed to 37°C. Mitochondria were incubated with deoxyguanosine triphosphate, dCTP, deoxythymidine triphosphate, and 20 μCi of [α-^32^P]-dATP for 2 hours at 37°C with gentle rotation. After incubation, the mitochondria were pelleted at 8000*g* for 4 min and washed twice with washing buffer [10% glycerol, 10 mM tris-HCl (pH 6.8), and 0.15 mM MgCl_2_]. Next, DNA was extracted using the Puregene DNA Purification Kit (Qiagen) according to the manufacturer’s instructions. DNA was resuspended in hydration buffer and incubated at 42°C for 1 hour. Samples were split into two groups to load them directly, after heat denaturation (5 min at 95°C), or after digestion with SacI-HF (New England Biolabs). All samples were then run on a 0.8% agarose gel (15 to 16 hours at 30 V) without ethidium bromide. Southern blotting was performed using standard protocols. Membranes were exposed to a phosphor screen for signal detection. To verify equal mitochondrial input, an aliquot of the mitochondrial suspension was collected before DNA extraction, separated by SDS–polyacrylamide gel electrophoresis (SDS-PAGE), and transferred to a polyvinylidene difluoride membrane. Membranes were probed with an antibody against VDAC1/Porin/VDAC3 (ab14734, Abcam) as a mitochondrial loading control.

### OXPHOS enzyme activities

Spectrophotometric assays were used to quantify the enzymatic activities of mitochondrial respiratory chain complexes, as previously described (*68*). Briefly, mitochondria were isolated and resuspended in assay buffer containing 250 mM sucrose, 15 mM magnesium acetate, 2 mM EDTA, BSA (0.5 g/liter), and 15 mM potassium dihydrogen phosphate (pH 7.2), adjusted to a final protein concentration of 1 mg/ml. Enzymatic activities of complexes I, I + III, II, II + III, and IV were measured using an Indiko Clinical Chemistry Analyzer following standard protocols. Activities were normalized to citrate synthase activity.

### RNA isolation and qPCR

Total RNA was extracted from 50 to 100 mg of frozen heart tissue using TRIzol Reagent (Ambion) and Lysing Matrix D tubes (MP Biomedicals). Tissues were homogenized in 1 ml of TRIzol using a FastPrep homogenizer (MP Biomedicals) at speed 6 for 20 s. Homogenized samples were incubated for 5 min at room temperature, followed by addition of 200 μl chloroform per 1 ml of TRIzol. Samples were vortexed briefly, incubated for 2 to 3 min, and centrifuged at 12,000*g* for 15 min at 4°C. The aqueous phase was transferred to a new RNase-free tube, mixed with 0.5 ml of isopropanol, and incubated at −20°C for 1 to 2 hours. RNA was pelleted by centrifugation at 12,000*g* for 10 min at 4°C and washed with 1 ml of cold 75% ethanol. Pellets were air-dried and resuspended in nuclease-free water. RNA concentration was determined using a Nanodrop spectrophotometer. cDNA was synthesized from 1 to 2 μg of total RNA using the High-Capacity cDNA Reverse Transcription Kit (Invitrogen), following the manufacturer’s protocol.

Gene expression was quantified using TaqMan gene expression assays and the TaqMan Universal PCR Master Mix II containing uracil-*N*-glycoslyase (Applied Biosystems) on a Quant Studio 6 Flex. qPCR was carried out in technical triplicates using a 384-well plate format. Reactions contained 10 ng of cDNA per well. Relative expression was calculated using the ΔCt method, normalized to housekeeping genes. Probes were selected from predesigned TaqMan Gene Expression Assays (Thermo Fisher Scientific). Markers used included *Mthfd2* (mm00485276), *Nppa* (mm01255747), and *Myh6* (mm00440359). *Actb* (mm01205647) was used as a loading control.

### RNA-seq library preparation, sequencing, and analysis

RNA-seq library preparation and sequencing were performed by Novogene. Briefly, mRNA was enriched from total RNA using poly-T oligo–attached magnetic beads. Following fragmentation, cDNA libraries were generated by first- and second-strand cDNA synthesis, end repair, A-tailing, adapter ligation, size selection, amplification, and purification. Library quality was assessed by Qubit, real-time PCR, and Bioanalyzer analysis before Illumina sequencing (Illumina NovaSeq X Plus). Raw sequencing reads were processed using fastp to remove adapter-containing reads, poly-N–containing reads, and low-quality reads. Clean reads were aligned to the mouse reference genome (mm39/GRCm39) using HISAT2 v2.2.1. Gene-level counts were generated using featureCounts v2.0.6. Differential expression analysis was performed using DESeq2 v1.42.0, with Benjamini-Hochberg correction for multiple testing.

For mutation-burden–resolved RNA-seq analyses, normalized gene expression values were correlated with total mtDNA mutation burden across individual *Polg*^hMut^ hearts. Genes were ranked by their Spearman correlation coefficient, and gene set enrichment analysis was performed using MSigDB Hallmark, and Gene Ontology Biological Process gene sets. Positive NES indicate pathways enriched among genes positively associated with total mtDNA mutation burden, whereas negative NES indicate pathways enriched among genes negatively associated with mutation burden.

### Extraction and sample clean-up for LC-MS–based nucleotide analysis from mouse heart tissue

Nucleotides were extracted and analyzed using a modified published method. Briefly, approximately 50 mg of deep-frozen mouse heart tissue was ground to a fine powder using a ball mill (Retsch MM400). Frozen tissue powder was extracted with 900 μl of ice-cold 10% trichloroacetic acid in UPLC-grade water (Biosolve) containing ^13^C_10_^15^N_5_ ATP and ^15^N_5_ AMP (100 ng/ml; MedChemExpress). Samples were mixed for 15 min at 4°C using an orbital shaker (Eppendorf), followed by centrifugation at 21,000*g* for 15 min at 4°C to pellet insoluble material.

The cleared supernatant was transferred to a fresh tube and mixed with 900 μl of 60% dichloromethane (Sigma-Aldrich) and 40% trioctylamine (Sigma-Aldrich). Samples were incubated for 10 min on an orbital mixer at 15°C and centrifuged for 10 min at 5000*g* at 15°C. From the phase-separated extract, 650 μl of the upper phase was collected and mixed with 900 μl of 0.5% acetic acid in UPLC-grade water. For LC-MS–based nucleotide analysis, samples were cleaned using anion-exchange solid-phase extraction columns (Strata-X-AW, 33 μm; Phenomenex), as described previously (*69*). Purified samples were analyzed by LC-MS as previously described (*70*).

### Oxidative damage assessment

Total lipid and protein fractions were isolated from mouse hearts as previously described (*71*). Briefly, hearts were homogenized in the presence of 1 mM butylated hydroxyanisole/butylated hydroxytoluene, and DNA was removed by streptomycin precipitation. Total lipids were extracted into chloroform, whereas total protein was isolated by deoxycholate/trichloroacetic acid precipitation and stored at −80°C until further analysis. For protein-based assays, the isolated protein pellets were solubilized in 50 mM NaOH and protein concentration was determined by the Pierce BCA Protein Assay Kit. Protein-bound MDA levels were measured fluorometrically in the isolated protein fraction using a specific thiobarbituric acid assay, with excitation/emission wavelengths of 535/550 nm (*72*). Protein carbonylation was assessed in the total protein fraction using the Protein Carbonyl Fluorometric Assay Kit (Cayman Chemical), based on the rhodamine B hydrazide assay. Fluorescence was measured using a spectrofluorometer rather than a plate reader, with excitation at 560 nm and maximum emission recorded at 585 to 595 nm. Total heart lipids were solubilized in 350 μl of 100% methanol, and lipid hydroperoxides were measured spectrophotometrically at 560 nm using a previously published method (*72*).

### Immunoblot analysis

Frozen heart tissue was homogenized using a TissueLyser (Qiagen) and resuspended in T-PER Tissue Protein Extraction Reagent (Thermo Fisher Scientific) supplemented with protease and phosphatase inhibitors (cOmplete EDTA-free Protease Inhibitor Cocktail and PhosSTOP, Sigma-Aldrich/Merck). Protein lysates were centrifuged, and the supernatant was used to determine protein concentrations using the Qubit Protein Assay. Equal amounts of protein were separated by SDS-PAGE and transferred to nitrocellulose membranes using an iBlot dry transfer system. Membranes were stained with Ponceau S to assess protein transfer and loading, blocked in 5% milk or 5% BSA in TBS-T, and incubated overnight at 4°C with primary antibodies against phospho-AMPK (Cell Signaling Technology, 2535, 1:1000 in 5% BSA/TBS-T), total AMPK (Cell Signaling Technology, 2532, 1:1000 in 5% BSA/TBS-T), phospho-ACC (Abcam, ab68191, 1:1000 in 5% milk/TBS-T), total ACC (Abcam, ab45174, 1:1000 in 5% milk/TBS-T), and β-actin/ACTB (Abcam, ab8226, 1:2000 in 5% milk/TBS-T). After washing, membranes were incubated with appropriate horseradish peroxidase–conjugated secondary antibodies, and signals were detected by chemiluminescence. Band intensities were quantified using Fiji. Phospho-AMPK and phospho-ACC signals were normalized to total AMPK and total ACC, respectively. β-Actin/ACTB and Ponceau S staining were used as loading and transfer controls.

### Cardiomyocyte isolation and live-cell imaging

Animals were euthanized by cervical dislocation, and hearts were immediately ex vivo perfused using the gentleMACS Octo Dissociator with Heaters, gentleMACS Perfusers 2, and the Heart Perfusion Kit (Miltenyi Biotec) according to the manufacturer’s instructions. Cell suspensions were filtered through a 100-μm cell strainer, and cardiomyocytes were allowed to sediment by gravity. The supernatant (containing heart immune residue cells) was collected, and the cardiomyocyte pellet was subjected to gradual calcium reintroduction at room temperature using four sequential transfer buffers containing increasing Ca^2+^ concentrations (0.06, 0.24, 0.5, and 1.0 mM; Table 1). At each step, 2 ml of transfer buffer were added, cells were allowed to sediment, and the supernatant was removed before proceeding to the next buffer. Cell quality after isolation and calcium reintroduction was assessed visually by light microscopy based on the presence of rod-shaped cardiomyocytes.

**Table 1.** Composition of buffers used for gradual calcium reintroduction in isolated cardiomyocytes. Transfer buffers were prepared from buffers A and B as indicated. Glucose, 2,3-butanedione monoxime (BDM), and BSA were added fresh to buffers A and B before pH adjustment at 37°C, and all buffers were maintained at 37°C before use.

Buffer	Components
Buffer A	135 mM NaCl, 4 mM KCl, 1 mM MgCl_2_, 10 mM Hepes, 0.33 mM NaH_2_PO_4_, 5.5 mM glucose, 10 mM BDM, and BSA (5 mg/ml) (pH 7.4) at 37°C
Buffer B	137 mM NaCl, 5.4 mM KCl, 0.5 mM MgCl_2_, 10 mM Hepes, 5.5 mM glucose, and 1.8 mM CaCl_2_ (pH 7.4) at 37°C
Transfer buffer 1	0.067 ml of buffer B into 1.93 ml of buffer A [final (Ca^2+^) 0.06 mM]
Transfer buffer 2	0.27 ml of buffer B into 1.73 ml of buffer A [final (Ca^2+^) 0.24 mM]
Transfer buffer 3	0.56 ml of buffer B into 1.44 ml of buffer A [final (Ca^2+^) 0.5 mM]
Transfer buffer 4	1.1 ml of buffer B into 0.9 ml of buffer A [final (Ca^2+^) 1 mM]

Following calcium reintroduction, cardiomyocytes were resuspended in prewarmed plating medium [M199 ×1 medium without phenol red supplemented with 5% fetal bovine serum, BDM (10 nmol/liter), and penicillin (100 U/ml)/streptomycin 100 μg/ml)], seeded at approximately 60% confluency onto laminin-coated black-wall, clear-bottom 96-well plates and incubated at 37°C in 5% CO_2_ for approximately 1 hour to allow attachment. The plating medium was then replaced with culturing medium [M199 ×1 medium without phenol red supplemented with 0.1% BSA, BDM (10 nmol/liter), penicillin (100 U/ml)/streptomycin (100 μg/ml), and 1× ITS-G].

For mitochondrial membrane potential measurements, cardiomyocytes were incubated with 100 nM Image-iT TMRM (Invitrogen) and Hoechst stain (1 μg/μl) for 30 min at 37°C and 5% CO_2_. Where indicated, carbonyl cyanide *m*-chlorophenyl hydrazone (20 μM in dimethyl sulfoxide) was added for an additional 15 min. Images were acquired using the ImageXpress High-Content Screening System (Molecular Devices) and analyzed using the IN Carta Image Analysis Software (Molecular Devices).

### Flow cytometry analysis of cardiac immune cells

Cardiac immune cells were analyzed from the noncardiomyocyte fraction obtained during cardiomyocyte isolation. Briefly, after gravity sedimentation of cardiomyocytes, the supernatant containing noncardiomyocyte cardiac cells was collected, filtered, washed, and stained for flow cytometry for 30 min at room temperature. Viability was assessed using Zombie Aqua Fixable Viability Dye, and CountBright Plus Absolute Counting Beads (Thermo Fisher Scientific) were added to estimate absolute cell numbers. Samples were acquired on a Sony ID7000 spectral flow cytometer and analyzed using ID7000 analysis software. Gating was performed to exclude debris, doublets, and dead cells, followed by identification of immune-cell populations among live single cells. Antibodies and staining reagents used for flow cytometry are listed in Table 2.

**Table 2.** Flow cytometry antibodies and staining reagents.

Marker/reagent	Fluorophore	Clone	Dilution	Supplier	Catalog no.
CD45	APC-Fire 810	30-F11	1:400	BioLegend	103174
CD3	BV605	17A2	1:100	BioLegend	100237
CD4	RY610	GK1.5	1:100	BD Biosciences	571146
CD8a	RY703	53-6.7	1:100	BD Biosciences	571447
CD19	APC	1D3	1:100	BD Biosciences	550992
CD11b	RB705	M1/70	1:100	BD Biosciences	570292
F4/80	BV421	BM8	1:100	Thermo Fisher Scientific	404-4801-82
MHC-II	Super Bright 780	M5/114.15.2	1:100	Thermo Fisher Scientific	78-5321-82
CD11c	PE	N418	1:80	BioLegend	117308
CD161/NK1.1	BV711	PK136	1:20	Thermo Fisher Scientific	407-5941-82
Ly6G	R718	1A8	1:80	BD Biosciences	567039
CD31	PE-Cy7	390	1:100	BioLegend	102418
Zombie Aqua Fixable Viability Dye	Zombie Aqua	-	1:2000	BioLegend	423102

### Multiplex cytokine measurements

Serum cytokine, chemokine, and growth factor concentrations were measured by Eve Technologies Corporation (Calgary, Alberta, Canada) using the Mouse Cytokine/Chemokine 68-Plex Discovery Assay Array (MD68). The assay was performed using Luminex xMAP technology on a Luminex 200 system with Bio-Plex Manager software, according to the manufacturer’s instructions. The panel was based on the MILLIPLEX Mouse Cytokine/Chemokine Magnetic Bead Panel (MCYT1-190K; MilliporeSigma) and quantified 68 analytes. Assay sensitivities ranged from 0.13 to 78 pg/ml, depending on the analyte.

### Tissue preparation and cryosectioning

Heart tissue was dissected from *Polg*^wMut^ and *Polg*^hMut^ mice and embedded in optimal cutting temperature (OCT) compound, and the OCT blocks were immediately frozen in liquid nitrogen–cooled isopentane. Hearts were oriented with the apex directed downward to obtain transverse cryosections perpendicular to the long axis of the heart. Sections were cut at a thickness of 5 μm using a cryostat (Thermo Fisher Scientific) and mounted onto poly-l-lysine–coated slides.

### COX/SDH histochemical staining

To assess mitochondrial respiratory chain activity COX/SDH staining was performed. In-plane matched cryosections were incubated for 30 min at 37°C in freshly prepared COX staining solution containing 100 μM cytochrome c, 4 mM 3,3-diaminobenzidine tetrahydrochloride, catalase (20 μg/ml), and 100 mM phosphate buffer (pH 7.0). Following three washes with phosphate-buffered saline (PBS), slides were incubated for 20 min at 37°C in freshly prepared SDH staining solution containing 130 mM sodium succinate, 200 μM phenazine methosulfate, 1 mM sodium azide, 1.5 mM nitroblue tetrazolium, and 100 mM phosphate buffer (pH 7.0). After three additional PBS washes, sections were dehydrated through graded ethanol (70, 95, and 99%) and mounted in xylol-containing mounting medium for bright-field microscopy (Zeiss AxioImager).

### Histological assessment (Masson’s trichrome staining)

In-plane matched cryosections were fixed in 10% neutral-buffered formalin for 1 hour at room temperature and subsequently incubated in Bouin’s fixative overnight. Sections were stained using the Masson’s Trichrome Stain Kit (Polysciences, 25088) according to the manufacturer’s instructions. After staining, sections were dehydrated through a graded ethanol series, cleared in xylene, and mounted using xylene-based mounting medium. Bright-field images were acquired using a Zeiss AxioScan.Z1 Slide Scanner. Collagen depositions are visualized by blue staining, whereas muscle and cytoplasmic structures stained red and nuclei stained black. Brightness and contrast were adjusted for better visualization.

### Immunofluorescence staining for CD45, WGA, and periostin

In-plane matched cryosections were fixed in 4% PFA/PBS for 10 min, washed, and blocked with 3% goat serum in 0.2% PBST (Triton X-100 0.2% in PBS) for 30 min at room temperature. Sections were incubated overnight at 4°C with rat anti-mouse CD45 antibody (Bio-Rad, MCA1031SBV515) or anti-rabbit Periostin (Abcam, ab14041) diluted 1:100 in blocking buffer. After washing, sections were incubated with Alexa Fluor 488–conjugated secondary antibody for 1 hours at room temperature, followed by nuclear staining with 4′,6-diamidino-2-phenylindole. Wheat-germ-agglutin conjugated to Alexa Fluor 488 (Invitrogen) was applied for 1 hour at room temperature. Slides were mounted with ProLong Diamond Antifade Mountant, and fluorescence images were captured using a Zeiss AxioImager microscope. Brightness and contrast were adjusted for better visualization.

### Transmission electron microscopy

Hearts were perfused by intracardiac injection of 4% formaldehyde. Heart tissue was subsequently dissected followed by immersion in a mixture of glutaraldehyde and paraformaldehyde in 0.1 M phosphate buffer. While submerged in fixative, the tissue was cut into smaller pieces (∼2 mm) and incubated at room temperature for approximately 1 hour then stored at +4°C until further processed. The fixed samples were first rinsed in 0.1 M phosphate buffer followed by postfixation in 2% fosmium tetroxide (Ted Pella, Redding, CA.) in 0.1 M phosphate buffer (pH 7.4) at 4°C for 2 hours. After stepwise dehydration in ethanol and en-bloc staining using 0.5% uranyl acetate (TAAB, Redding, England), the samples were acetone dehydrated before resin infiltration and finally embedded in LX-112 (Ladd Research, Essex Junction, VT). Ultrathin sections (∼100 nm) were prepared using an EM UC7 (Leica microsystems, Wetzlar, Germany) and transferred onto formvar-coated slot grids (Ted Pella, Redding, CA) and contrasted with uranyl acetate (TAAB, Redding, England) followed by lead citrate (Merck, Darmstadt, Germany). The grids were examined in an HT7800 transmission electron microscope (Hitachi High-Technologies, Krefeld, Germany) at 100 kV, and digital images were acquired using a 20MPx Xarosa complementary metal-oxide semiconductor camera (EMSIS GmbH, Münster, Germany).

### Statistical analysis

The sample size and statistical tests performed are indicated in the figure legends. Statistical analysis and generation of graphs were performed with GraphPad Prism v10, except for quantitative MS data, which were analyzed and plotted using R Studio, Instant Clue v.0.10.10. and Flaski version 3.20.0 (https://flaski.age.mpg.de/about/) as described above. Values of *P* < 0.05 were considered statistically significant.
