# Supplementary material for: Cardiomyocyte-intrinsic somatic mtDNA mutations induce an OXPHOS-dependent immune response and promote progressive heart failure
Source: Sci Adv. 2026 Sep 4;12(36):eaec8606. doi: 10.1126/sciadv.aec8606 (PMC13544208; doi:10.1126/sciadv.aec8606)
Supplement: Supplementary file 1 [file sciadv.aec8606_sm.pdf]

Science Advances
AAAS

Supplementary Materials for

Cardiomyocyte-intrinsic somatic mtDNA mutations induce an OXPHOS-dependent immune response and promote progressive heart failure

Kristina Bubb *et al.*

Corresponding author: Nils-Göran Larsson, nils-goran.larsson@ki.se

Sci. Adv. **12**, eaec8606 (2026)
DOI: 10.1126/sciadv.aec8606

This PDF file includes:

Figs. S1 to S9

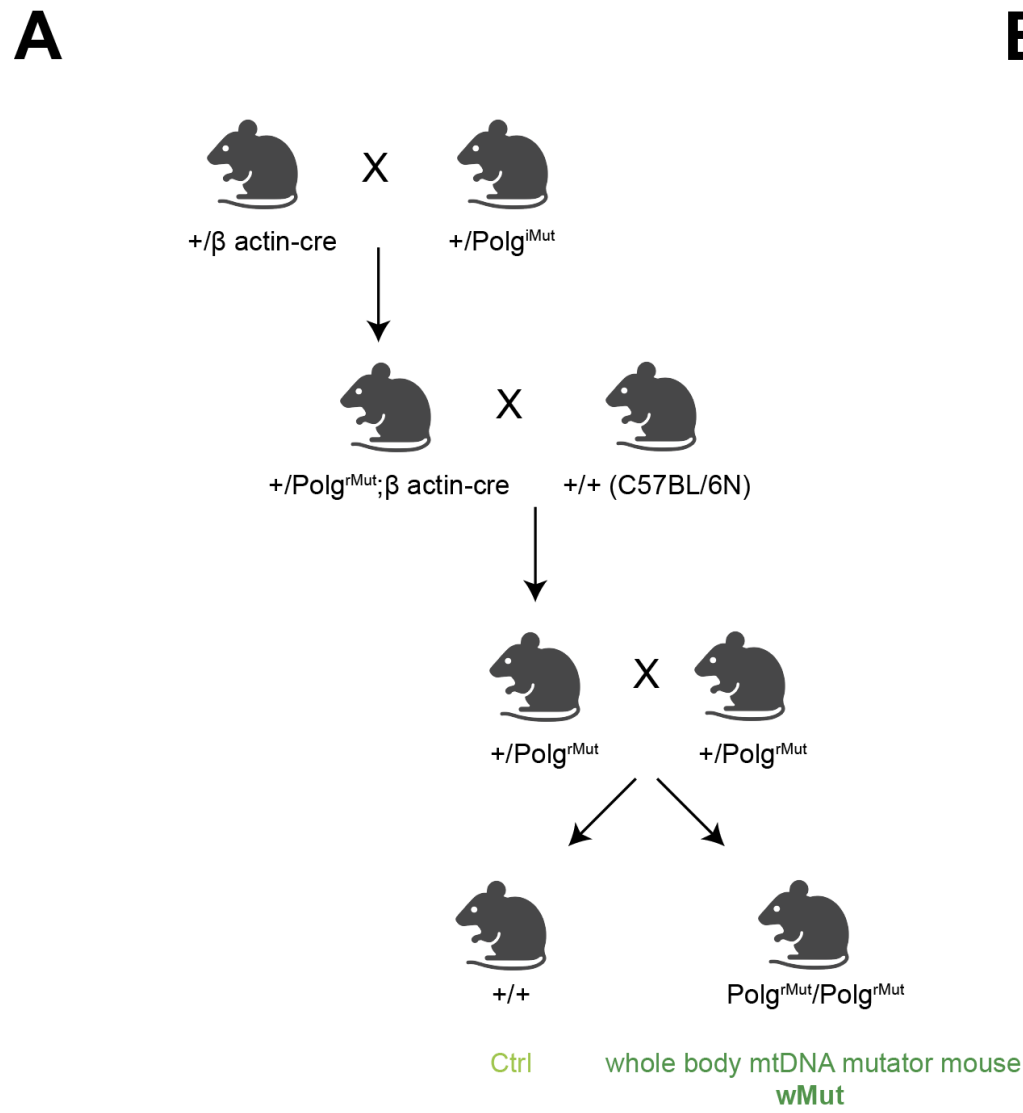

Fig. S1.

Schematic representation of mating strategies (A) Mating strategy to create *Polg*^{wMut} mice (wMut) with ubiquitous POLG^{D257A} expression. Heterozygous *Polg*^{iMut} mice were crossed with +/β-actin-cre transgenic mice to induce ubiquitous cre-mediated recombination of the *Polg*^{iMut} to generate *Polg*^{rMut} mice. The *Polg*^{rMut} allele was transmitted through the germline and the β-actin-cre transgene was remove by breeding. Mice homozygous for the *Polg*^{rMut} allele are denoted *Polg*^{wMut} mice. **(B)** *Polg*^{iMut} mice were crossed with Ckmm-cre mice to *Polg*^{hMut} mice with tissue-specific POLG^{D257A} expression. Graphical elements were created with BioRender.com (Larsson, N. 2026; https://BioRender.com/fdh3wkr)

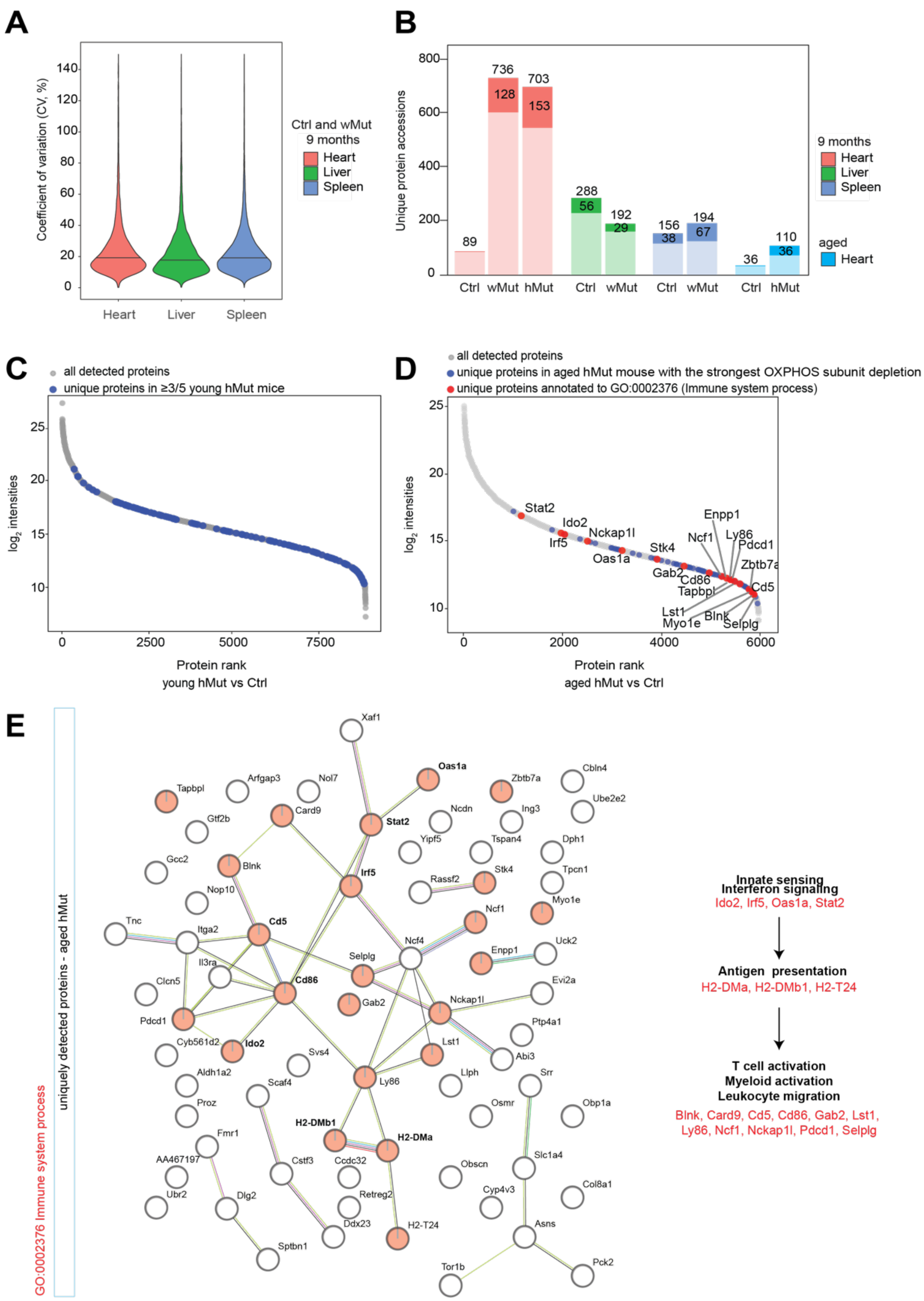

Fig. S2.

Quality control and protein detection of the proteomic datasets (A) Coefficient of variation plot of detected protein numbers in heart, liver, and spleen tissue from 9-month-old control and *Polg*^{wMut} mice. **(B)** Bar plot showing the absolute numbers of uniquely detected protein accessions per dataset. The fraction of proteins detected in at least three samples is indicated as a proportion

of each bar. **(C–D)** Protein rank plots displaying log_2-transformed maximum protein intensities for 9-month-old (C) and aged (D) *Polg*^{hMut} hearts. Proteins uniquely detected in each comparison (as shown in Figures 5C and S2E) are highlighted in blue. Proteins annotated with GO:0002376 ("Immune system process") are indicated in red and annotated with gene names. **(E)** STRING database network of proteins uniquely detected in aged *Polg*^{hMut} heart exhibiting the strongest reduction in OXPHOS subunit abundance. Proteins highlighted in red are annotated with GO:0002376 (Immune system process). Proteins discussed in the text are highlighted in bold. Schematic representation of induced pathways is shown.

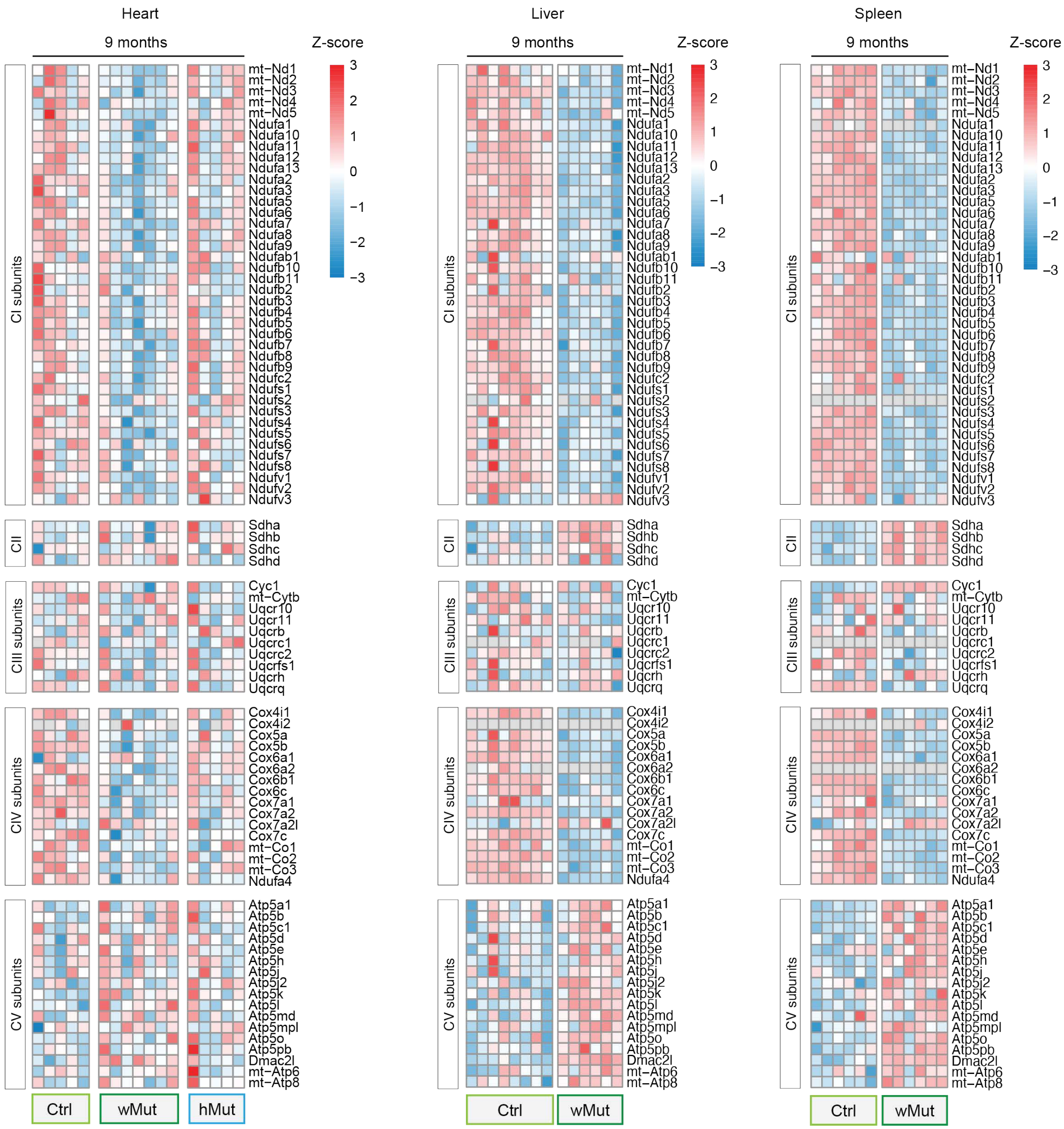

Fig. S3.

Tissue-specific effects of mtDNA mutations on OXPHOS subunits abundance Heatmaps show Z-score-normalized abundance levels of OXPHOS complex subunits in heart, liver, and spleen proteomes from 9-month-old control (Ctrl), *Polg*^{wMut} (wMut) and *Polg*^{hMut} mice (hMut).

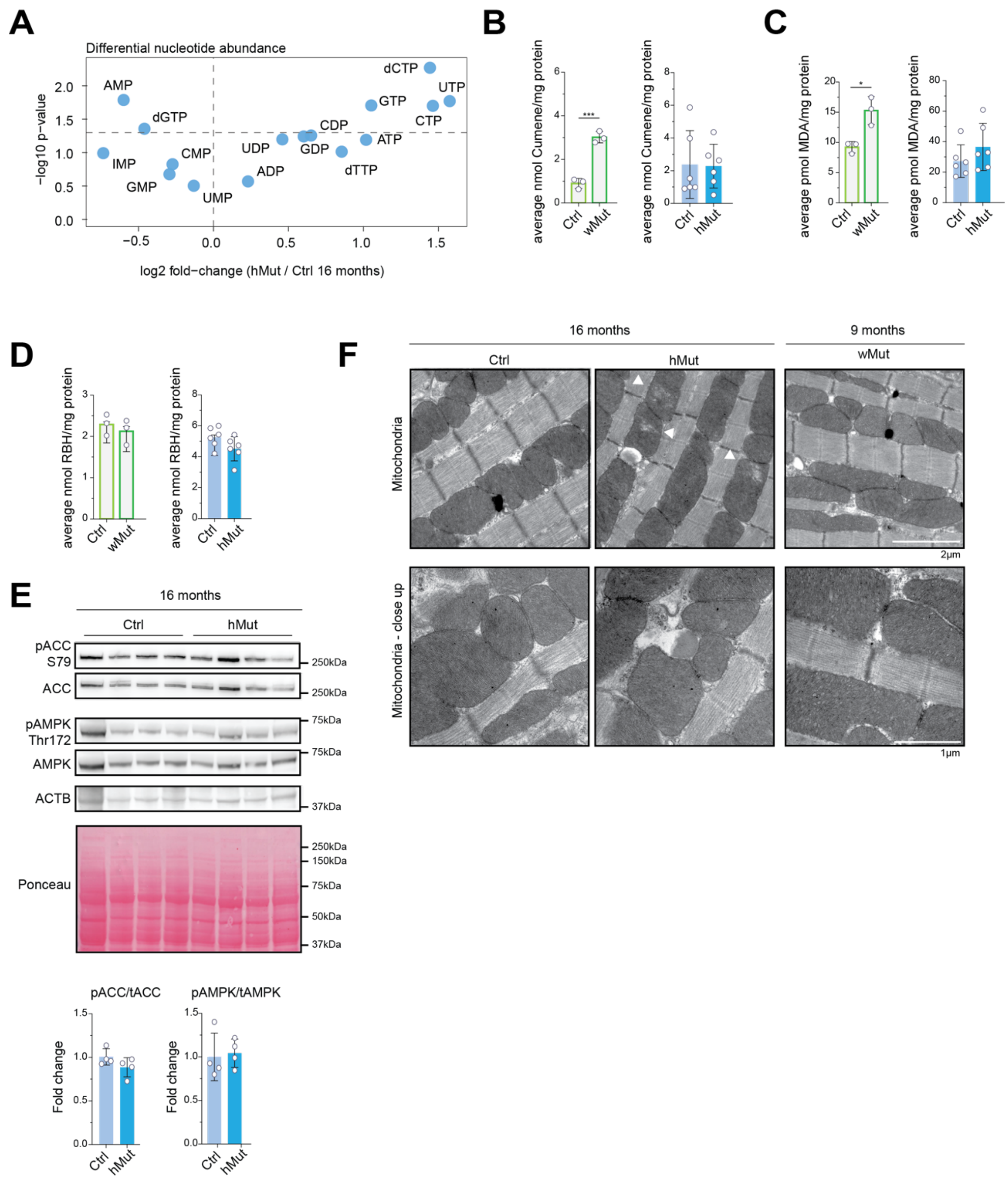

Fig. S4.

Assessment of nucleotide levels, oxidative damage, energetic stress signaling, and mitochondrial ultrastructure in aged *Polg*^{hMut} hearts.

(A) Targeted metabolomic analysis of cardiac nucleotide pools in 16-month-old control and *Polg*^{hMut} hearts. Volcano plot shows differential nucleotide abundance relative to controls. Each point represents one nucleotide. **(B–D)** Quantification of oxidative damage markers in 9-month-old control and *Polg*^{wMut} hearts and in 16-month-old control and *Polg*^{hMut} hearts. Lipid hydroperoxides are shown as nmol cumene hydroperoxide equivalents per mg protein **(B),** protein-bound malondialdehyde (MDA) as pmol MDA per mg protein **(C),** and protein carbonylation as

nmol reduced biotin hydrazide (RBH) per mg protein **(D)**. Data are presented as mean ± SD, unpaired two-tailed Welch's t-test; $*P < 0.05$, $***P < 0.001$. **(E)** Immunoblot analysis of AMPK and ACC phosphorylation in 16-month-old control and *Polg*^{hMut} hearts. Representative immunoblots and quantification of pAMPK/total AMPK and pACC/total ACC are shown. ACTB and Ponceau staining are shown as loading controls. **(F)** Transmission electron microscopy images of cardiac mitochondria from 16-month-old control and *Polg*^{hMut} hearts, and from 9-month-old *Polg*^{wMut} hearts. Occasional focal intramitochondrial regions with apparent cristae loss or disorganization are marked by arrowheads. Scale bars: 2 µm upper panel and 1 µm lower panel.

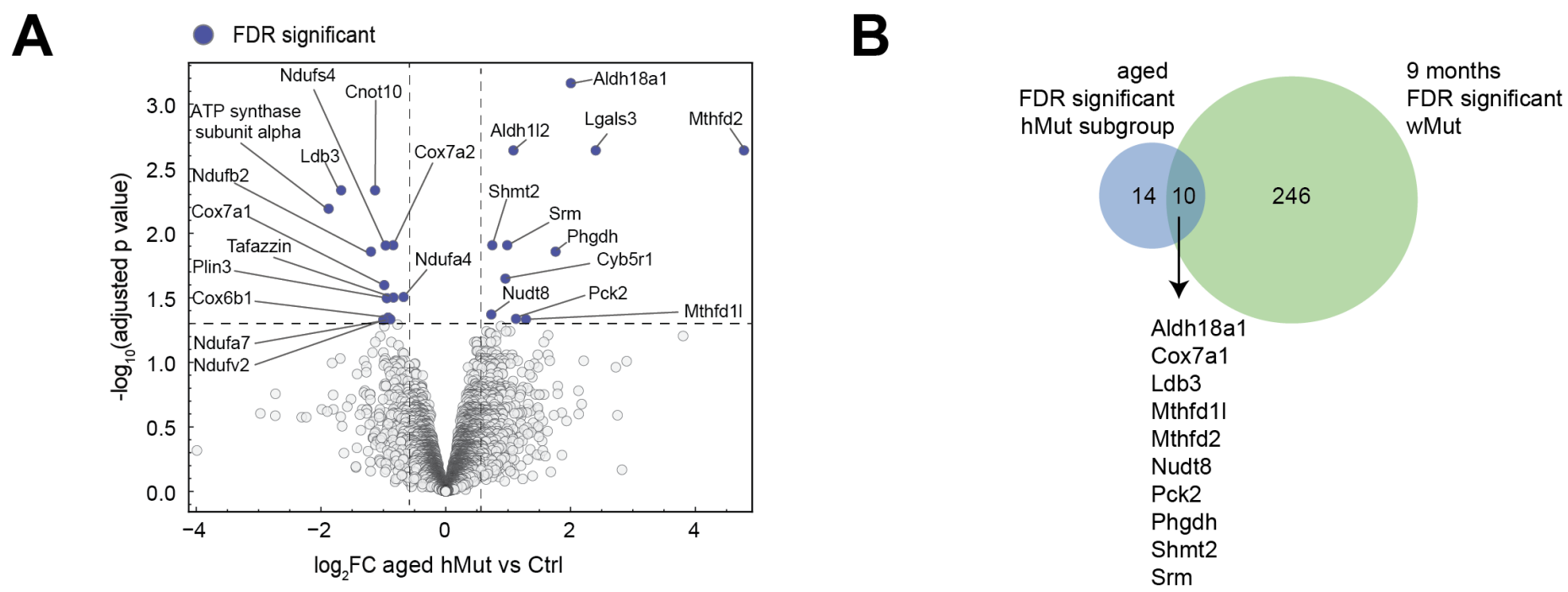

Fig. S5.

Proteomic analysis of a subgroup of OXPHOS subunits depleted aged *Polg*^{hMut} hearts (A) Volcano plot showing results of an adapted statistical analysis comparing control hearts to *Polg*^{hMut} hearts with depletion of OXPHOS subunits. Significantly altered proteins are highlighted in blue (-$\log_{10}$ adjusted p-value>1.3). **(B)** Venn diagram illustrating the overlap of significantly regulated proteins in aged *Polg*^{hMut} hearts, and 9-month-old *Polg*^{wMut} hearts.

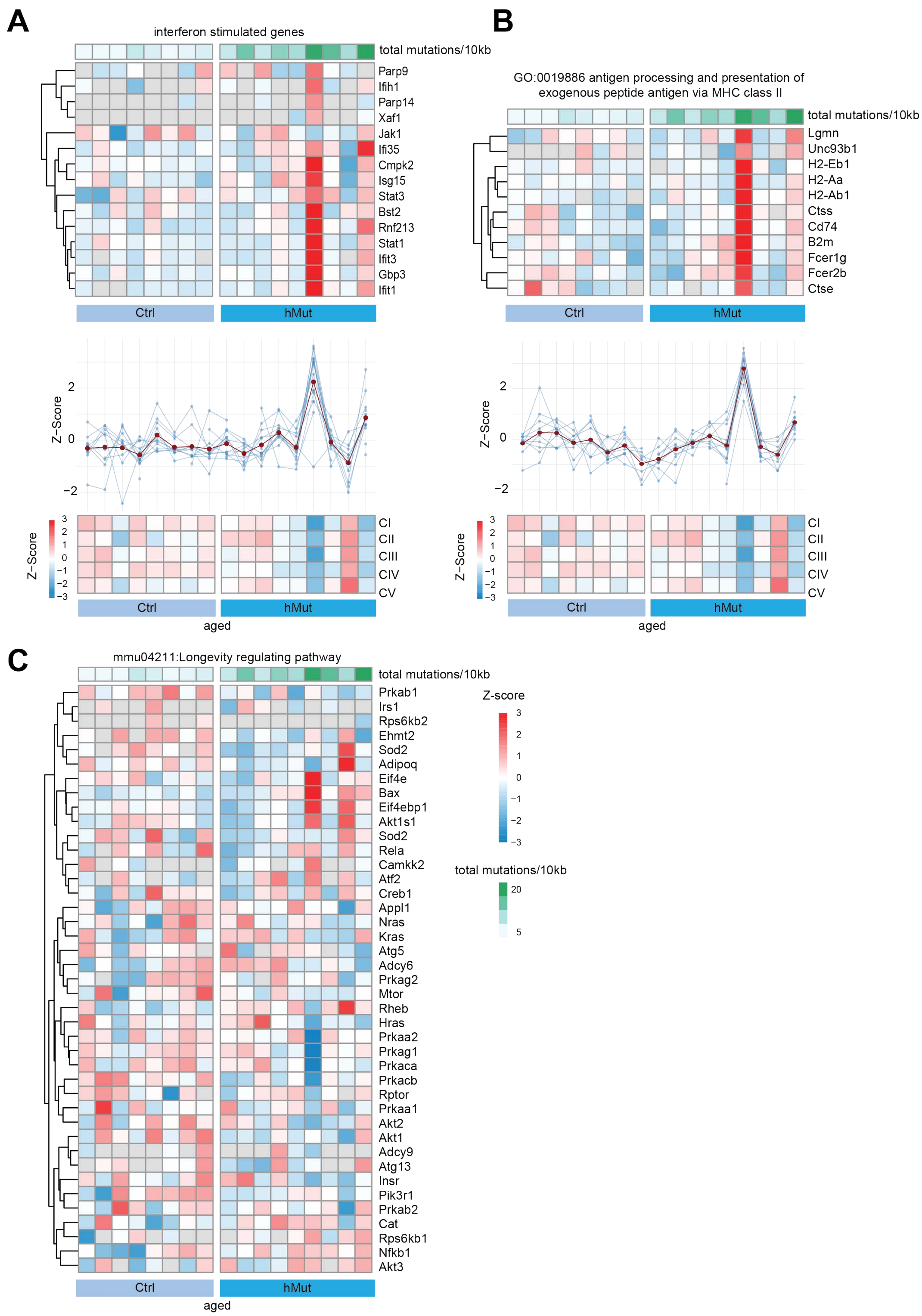

Fig. S6.

OXPHOS-dependent immune response and OXPHOS-independent ageing response in *Polg*^{hMut} hearts (A) Heatmap of interferon-stimulated genes in aged controls (Ctrl) and *Polg*^{hMut} hearts (hMut). Line plots display Z-scores for individual genes across all samples, with individual

protein abundance profiles in blue and the per-sample mean Z-score overlaid in red. Below, a heatmap of mean abundances for OXPHOS subunits I-V illustrates the relationship between OXPHOS subunit depletion and the coordinated regulation of proteins associated with these interferon-stimulated genes. **(B)** Heatmap of GO:0019886-associated proteins in aged controls and *Polg*^{hMut} hearts. Line plot shows Z-scores for individual genes within this pathway across individual samples, with individual profiles in blue and the mean per-sample Z-score in red. A heatmap of mean OXPHOS subunit I-V abundances are shown below to illustrate coordinated regulation relative to OXPHOS depletion. **(C)** Heatmap showing all detected KEGG mmu04211 longevity-associated proteins in aged control (Ctrl) and *Polg*^{hMut} (hMut) hearts. The top annotation indicates total mtDNA mutations per 10 kb. Heatmaps show row-wise Z scores. The Z-score and mutation-load legends shown in panel C apply to panels A-C.

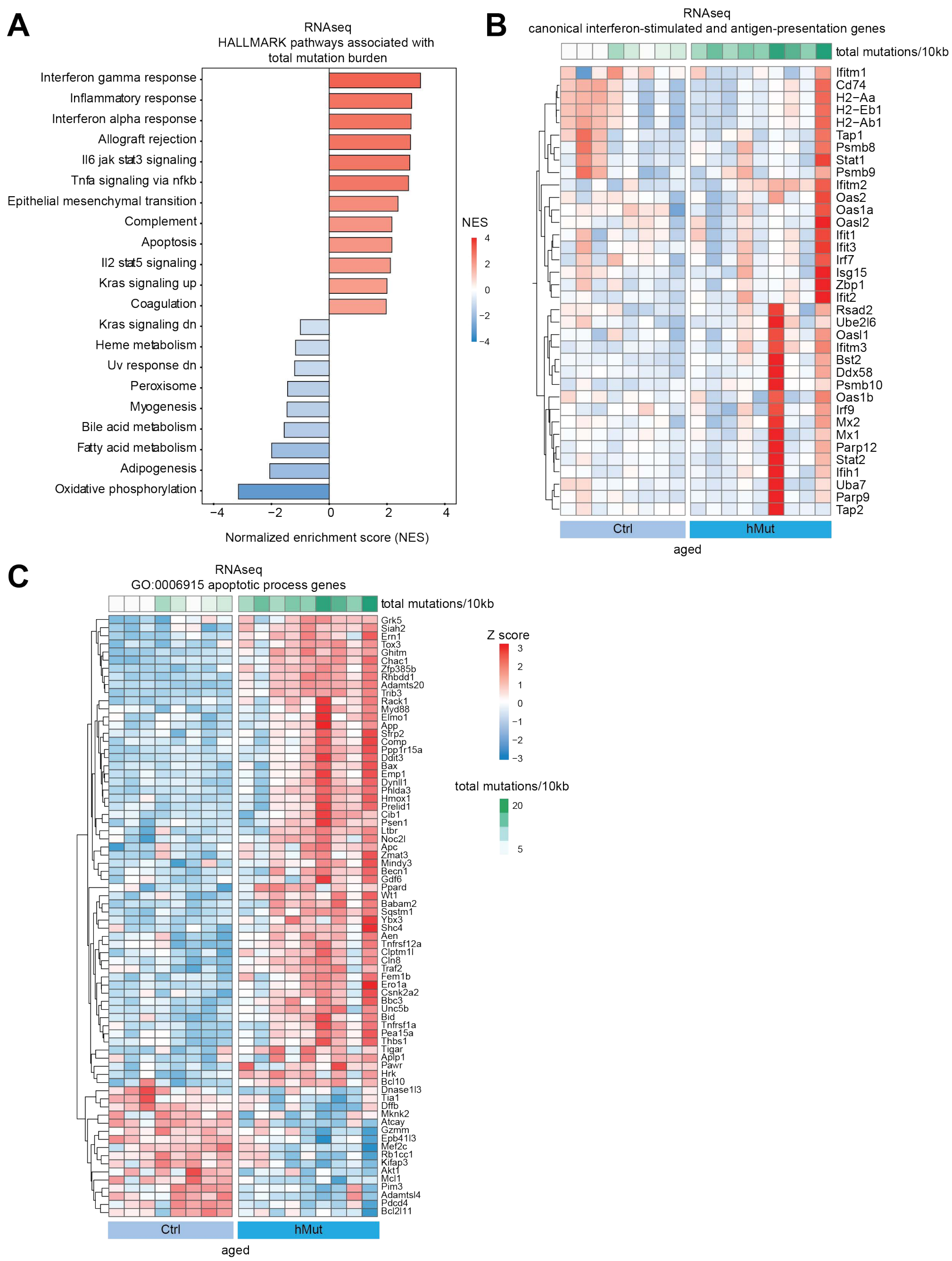

Fig. S7.

RNA-seq validation of mutation-burden-associated immune and stress-response activation in aged *Polg*^{hMut} hearts

(A) Hallmark gene set enrichment analysis of RNA-seq data from aged *Polg*^{hMut} hearts. Genes were ranked according to their Spearman correlation with total mtDNA mutation burden. Positive normalized enrichment scores (NES) indicate pathways enriched among genes positively associated with mutation burden, whereas negative NES indicate pathways enriched among genes negatively associated with mutation burden. **(B)** Heatmap showing expression of canonical interferon-stimulated and antigen-presentation genes in *Polg*^{hMut} RNAseq dataset. **(C)** Heatmap showing expression of genes annotated to apoptotic process (GO:0006915) in *Polg*^{hMut} RNAseq

data set. The top annotation indicates total mtDNA mutations per 10 kb. Heatmaps show row-wise Z scores. The Z-score and mutation-load legends shown in panel C apply to panels B-C.

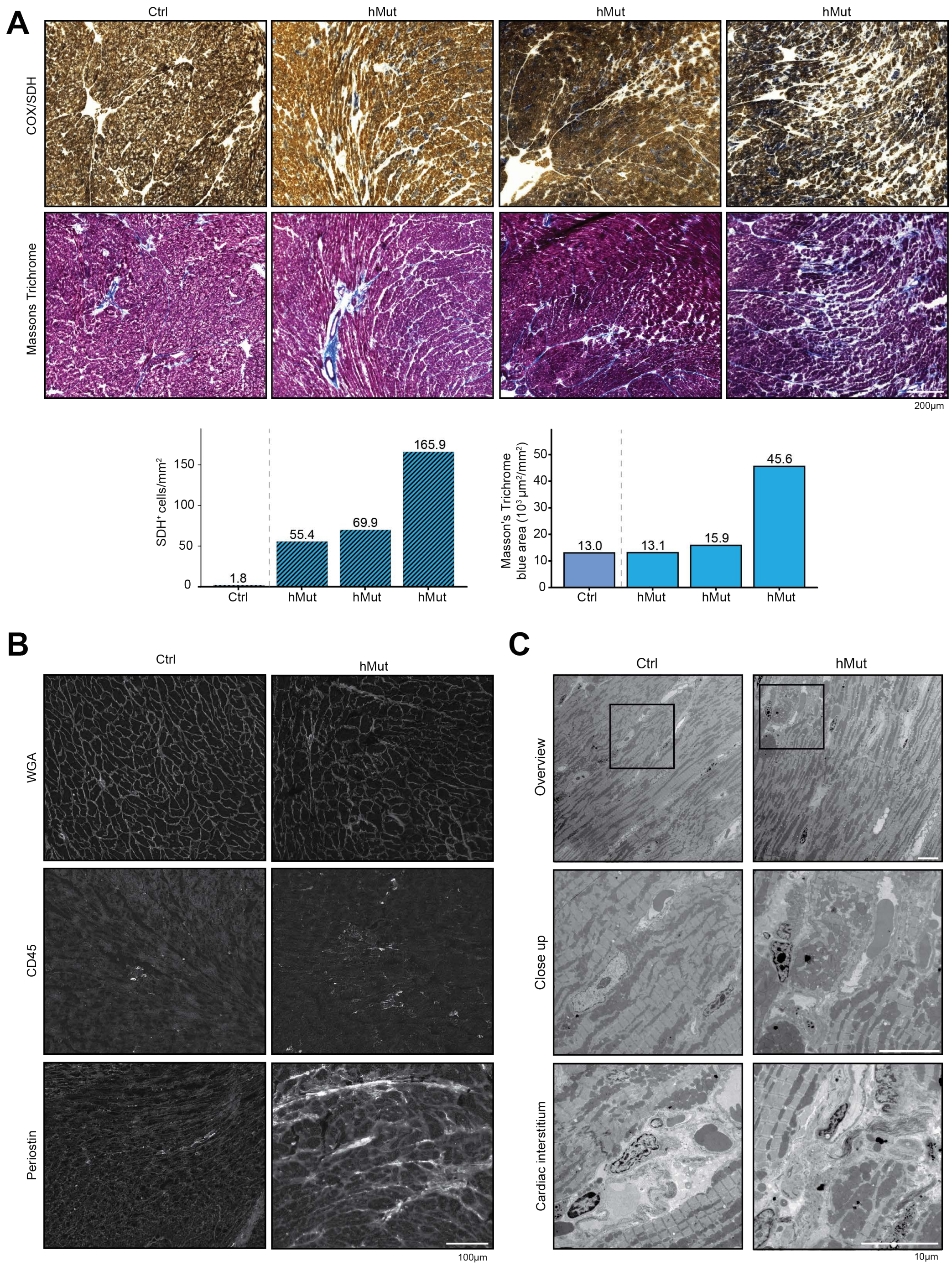

Fig. S8.

Local OXPHOS deficiency is associated with fibrotic remodeling and immune-cell infiltration in aged *Polg*$^{\mathbf{hMut}}$ hearts

(A) Representative cytochrome c oxidase/succinate dehydrogenase (COX/SDH) histochemistry and Masson's trichrome staining of consecutive cardiac sections from 16-month-old control and *Polg*^{hMut} hearts. Upper panels show COX/SDH staining, where SDH-positive cardiomyocytes (blue) indicate respiratory-chain deficiency. Lower panels show Masson's trichrome staining of adjacent sections, where blue staining indicates collagen deposition. The control (Ctrl) Masson's trichrome image and the second *Polg*^{hMut} Masson's trichrome image were generated from the same original stained images used for the corresponding panels in Fig. 6F. Bar graphs show quantification of SDH-positive cardiomyocytes and Masson's trichrome-positive collagen area in the corresponding sections. Scale bar: 200 µm. **(B)** Wheat germ agglutinin (WGA), CD45, and periostin staining of cardiac sections from 16-month-old control and *Polg*^{hMut} hearts. Scale bar: 100 µm. **(C)** Transmission electron microscopy images of 16-month-old control and *Polg*^{hMut} hearts. Overview images and higher-magnification close-ups show collagen deposition and immune-cell presence in the cardiac interstitium. Scale bars: 10 µm.

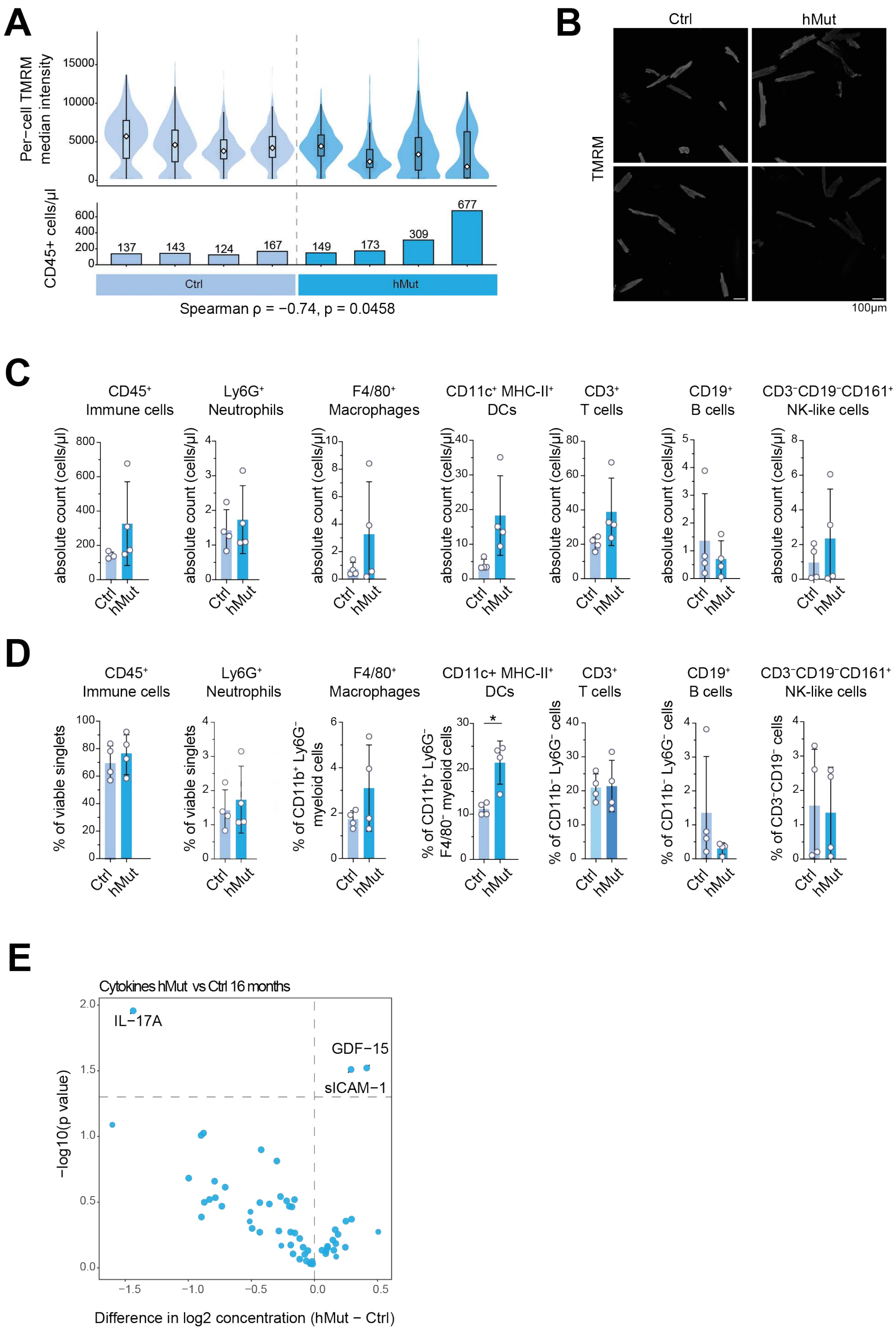

Fig. S9.
Reduced cardiomyocyte mitochondrial membrane potential is associated with cardiac immune-cell infiltration in aged *Polg*^{hMut} hearts.

(A) TMRM intensity in isolated cardiomyocytes from 16-month-old control and *Polg*^{hMut} hearts, shown together with cardiac CD45^{+} immune-cell abundance measured by flow cytometry in the corresponding animals. Violin plots show per-cell TMRM intensity distributions for each animal, with embedded boxplots indicating the median and interquartile range. Bar plots show cardiac CD45^{+} immune-cell abundance/µl in the corresponding animals. Spearman correlation analysis was performed using the animal-level median per-cell TMRM intensity and cardiac CD45^{+} immune-cell abundance (Spearman's $\rho = -0.74$, $p = 0.0458$). **(B)** Representative images of TMRM-stained cardiomyocytes isolated from 16-month-old control and *Polg*^{hMut} hearts. Scale bars: 100µm. **(C)** Flow-cytometric quantification of absolute immune-cell counts and **(D)** relative abundance in 16-month-old control and *Polg*^{hMut} hearts. Data are presented as mean ± SD, unpaired two-tailed Welch's t-test; $^{*}P < 0.05$ **(E)** Serum multiplex cytokine profiling in 16-months-old control and *Polg*^{hMut} mice. Volcano plot showing differences in $\log_2$ cytokine concentration between *Polg*^{hMut} and control mice.
